# Why Do Emergency Medical Service Employees (Not) Seek Organizational Help for Mental Health Support?: A Systematic Review

**DOI:** 10.3390/ijerph22040629

**Published:** 2025-04-17

**Authors:** Sasha Johnston, Polly Waite, Jasmine Laing, Layla Rashid, Abbie Wilkins, Chloe Hooper, Elizabeth Hindhaugh, Jennifer Wild

**Affiliations:** 1 Department of Experimental Psychology, University of Oxford, Oxford OX1 2JD, UK; 2 South Western Ambulance Service NHS Foundation Trust, Bristol BS16 IDE, UK; 3 Phoenix Australia Centre for Posttraumatic Mental Health, University of Melbourne, Carlton, VIC 3053, Australia

**Keywords:** mental health, emergency medical services, paramedic, organizational support, barriers, enablers, ambulance, psychology, culture

## Abstract

Emergency medical service (EMS) ambulance employees play a critical role in emergency healthcare delivery. However, work-related experiences can compromise their mental health and job satisfaction. Despite available supportive services offered by EMS organizations, employee uptake remains low, while mental ill health and suicide rates remain higher than those of the general population. Understanding barriers to and enablers of such support is crucial for addressing factors that connect employees with the services designed to help. This systematic review identified 34 relevant articles and utilized an innovative process of integrating quantitative and qualitative aspects of the primary and gray literature to provide a qualitative synthesis of barriers and facilitators as perceived by EMS employees. Themes of employee (in)ability to ask for help, tailored person-centered support, and education and training about mental health were overarched by organizational culture. Barriers included perceived organizational obligation rather than genuine care, alongside machismo and stigma. Enablers included valuing and acknowledging employee risk by providing time and normalizing support utilization at work. Reframing machismo from dominance, competition, and toughness to respect, perseverance, and courage; promoting adaptive coping; and providing time and training were essential. Future research should aim to understand the factors influencing employee utilization of supportive interventions based on these themes.

## 1. Introduction

The World Economic Forum projected that more than half of the global economic burden attributable to non-communicable diseases will be caused by mental ill health by 2030 [1]. Emergency medical service (EMS) ambulance employees, at the front line of healthcare, are particularly vulnerable, facing unique challenges such as exposure to critical incidents, long hours, and shift work. Due to these factors, mental health and well-being can be compromised by work-related traumatic events, as well as everyday stressors caused by increasing demand for EMS services and wider healthcare system failures, such as lengthy hospital handover and discharge delays [2]. Consequently, EMS organizations struggle to provide psychologically safe workplaces that promote employee well-being and actively prevent harm.

Suicide is a particular concern, with fifty-five English EMS employee suicides registered with the Association of Ambulance Chief Executives suicide register since data collection commenced in August 2018 [3]. Furthermore, Mars et al. [4] and Office for National Statistics data [5] identified a 75% increased suicide risk among male paramedics compared with the general population. Several risk factors contribute to mental ill health such as genetics, circadian rhythm disruption, loneliness, stressful life events, and physical ill health. Among EMS employees, alcohol and drug misuse and high rates of childhood adverse events, such as trauma, abuse, and neglect, have been identified [6,7]. Research by the mental health charity Mind found that EMS employees were twice as likely to identify problems at work as the main cause of their mental ill health, compared with the general workforce [7]. Almost nine in ten EMS employees reported symptoms of mental ill health. Some EMS organizations reported 50% of employees leaving the workforce, compromising organizational capability, with poor staff mental health and organizational culture cited as primary contributing factors [8,9].

In response, EMS organizations provide employees with a range of supportive wellness services such as counseling, mindfulness, and referral pathways to charitable and community-based interventions. However, evidence suggests that the uptake of such services is low, and employee satisfaction surveys have not improved despite investment in supportive services [10]. Organizational support is a distinct concept that reflects an organization’s dedication to its employees. It includes the principles of effort–reward expectations, job satisfaction, and procedural fairness. EMS employee well-being is positively associated with access to organizational support, which is important as such support can reduce symptom severity, prevent suicide, and enable people to thrive at work [11]. Thriving employees underpin patient care and safety [12,13,14].

In the context of this review, organizational support refers to assistance provided by the employing EMS organization for employee mental health and well-being. This includes commissioned employee assistance programs (EAPs); peer support networks set up by the organization; and targeted interventions like post-incident debriefing, mindfulness classes, and counseling. Existing systematic reviews emphasize the importance of organizational support for employee well-being yet often lack detailed examination of the barriers and facilitators from an employee perspective. They also tend to exclude the gray literature, limiting insights from materials such as opinion pieces and industry briefings that capture nuances peer-reviewed research may overlook. Given the low uptake of support designed to aid mental health and well-being for EMS employees, this study aimed to gain a broader perspective on the factors influencing why employees will or will not utilize organizational support. To understand these factors, we expanded on prior reviews by integrating diverse sources and applying updated methodologies to identify underlying factors influencing support utilization. Our strategy adopted a comprehensive approach, while reducing bias, offering a more nuanced understanding of organizational support in this context. We conducted a full mixed methods systematic review of the literature, including all methodologies and gray literature. In addition, we identify supportive interventions offered through or by EMS organizations to support employee mental health and well-being reported in the included articles. Interventions are summarized using a Template for Intervention Description and Replication (TIDieR) checklist, which is presented alongside a comprehensive synthesis of the perceived barriers and enablers influencing whether employees will use the support provided.

## 2. Methods

A protocol for this review has been published [15] and registered with the International Prospective Register of Systematic Reviews (PROSPERO; CRD42022299650).

### 2.1. Search Strategy

Developed in partnership with expert librarians (see Supplementary Material S1), our search criteria included quantitative, qualitative, and mixed-method studies written in English since 1st December 2004. This date accounted for changes in employee well-being legislation and policy introduced to worldwide EMS organizations following the 11th September 2001 USA terrorist attacks [16,17,18]. The reference lists of selected studies were hand-searched for further material for inclusion.

Searches, using keywords and relevant thesauri, rerun in January 2024, included the following databases: AMED, CINAHL, the Cochrane Central Register of Controlled Trials and the Cochrane Database of Systematic Reviews via the Cochrane Library, EMBASE, EMCARE, HMIC, MEDLINE, PsycINFO, Scopus, and Web of Science. To ensure all the available and relevant research was captured, we sought gray literature from the OpenGrey, MedNar, and ProQuest databases and through the webpages of industry and charitable organizations active in supporting EMS ambulance employee mental health (Supplementary Material S2, Table S1). We considered all article types, including systematic reviews and primary studies, to provide a comprehensive overview while avoiding data duplication by extracting and contextualizing the unique insights from each source. The search results are presented in a Preferred Reporting Items for Systematic Review and Meta-Analysis (PRISMA) flow diagram [19].

### 2.2. Eligibility Criteria

The searches were limited to organizational support for adults (18+) working for state/government commissioned out-of-hospital EMS ambulance organizations. We excluded EMS students, volunteers, friends or family, disaster response, intensive mobile units connected with specialist hospital services such as intensive care units, and private EMS workers as these groups were likely to be offered different or no support from EMS organizations. All EMS service job roles were included as evidence suggested that all could be affected by work-related experiences. We considered self-reported barriers to and facilitators of accessing and seeking organizational mental health support. Organizational support referred to assistance provided from EMS employers for employee mental health and well-being. This included commissioned EAPs; peer support networks set up by the organization; and targeted interventions such as post-incident debriefing, mindfulness classes, and counseling. We excluded informal social/family support, external agency support not commissioned by the employer (e.g., charitable organizations), and assistance from other health organizations such as general practitioners. Database papers were excluded if they (1) lacked relevance to the research question; (2) focused on patient- or population-based care; or (3) involved non-target personnel, such as in-hospital emergency department staff or police. Gray papers were primarily excluded for not addressing barriers to and facilitators of organizational support. For articles we could not access, we contacted the first authors using email addresses found through Google searches and www.researchgate.net.

Articles were imported into a Mendeley reference management system to remove duplicates and aid manual screening of titles and abstracts. One reviewer (SJ) independently reviewed all articles, and a second reviewer (AW) reviewed a random 10% sample (n = 479) from databases and registers and 10% (n = 18) from the gray literature. Both reviewers independently screened articles using a PICoT concept (see Supplementary Material S3, Table S2).

Studies meeting all four criteria advanced to full text review and data extraction. Disagreements were arbitrated by a third reviewer (KS). Kappa statistics for inter-rater agreement demonstrated a moderate to good level of agreement (κ > 0.60) [20].

### 2.3. Quality Assessment

Quality assessment of all included papers was independently completed by two reviewers (SJ and LR) based on design and reporting. Different checklists were applied depending on the type of article and methodology being assessed (see Appendix A, Table A1, for checklist items) [21,22,23,24]. Each paper received a quality assessment score ranging from 0 (not reported) to 2 (fully reported).

Differences in checklist application or item assessment between reviewers were resolved through discussion and third-person arbitration if required. For instance, any studies reported as “mixed methods” but only utilizing cross-sectional surveys with brief free-text responses were assessed using a quantitative tool as the descriptive data were deemed to be insufficient for qualitative analysis. The results were summarized in a color-coded table to highlight whether articles were assessed as being high, medium, or low risk for bias.

### 2.4. Data Extraction

Underpinned by a realist approach and reflexive journaling (see Supplementary Material S4) we created and piloted a data extraction template in Microsoft Excel. Two reviewers (SJ and JL) independently extracted key data from the results sections of the included papers and quotations relating to barriers to and enablers of organizational mental health support. The two reviewers discussed, debated, and resolved data extraction decision-making during regular Microsoft Teams meetings, with a third reviewer (KS) available to arbitrate any unresolved issues. Due to the lack of research and the complexities underpinning EMS employee perceptions, an open-minded inductive approach was chosen over a theory-driven deductive method for generating and exploring EMS employee perspectives. The two reviewers compared, debated, and resolved differences in decision-making about data extraction during weekly meetings. In addition, a 12-item Template for Intervention Description and Replication (TIDieR) checklist was populated to summarize interventions discussed in the included articles and to contextualize the review findings [25,26,27]. If data were missing or additional information was required, authors were contacted.

### 2.5. Data Synthesis

Following the Joanna Briggs Institute (JBI) methodology, a mixed-methods enquiry was undertaken to capture employee barriers to and enablers of organizational support for employee mental health [28]. This involved “qualitization” by transforming quantitative data into textual descriptions to represent numerical data, which were subsequently integrated with qualitative data and uploaded to NVivo 12 software. Two reviewers using an inductive approach agreed on a coding structure. Reviewer 1 (SJ) coded all data, while reviewer 2 (JL) independently coded 10% for comparison.

Pragmatic selective coding relevant to barriers to and enablers of ambulance employees utilizing organizational mental health support adopted a predominately semantic focus, supported by latent coding when needed, to produce a realist, descriptive account of the findings from each of the included papers. The rationale for the construction of any latent coding was recorded to aid transparency and reflexivity and to minimize researcher bias. During weekly meetings and regular email, the reviewers ensured the developing coding structure was appropriate and made sense in the context of the data. Once all data had been coded, the final codes were reviewed and refined and nominal labels agreed upon. A reflexive thematic analysis of the data was then undertaken.

### 2.6. Data Analysis

Using reflexive thematic analysis (RTA), two reviewers (SJ and JL) collaboratively constructed data through shared screen Microsoft Teams calls and reflexively through independent journaling. Decision-making was compared and discussed, while building and revising themes, enabling bracketing and challenge of bias. Codes were crafted into categories that represented perceived barriers, enablers, or both, within the organizational support context, aiding systematic theme construction and development based on patterns for overlap and similarities. Visual thematic and conceptual mapping functionality in NVivo was used to explore relationships.

All themes were reviewed and agreed upon by three authors (SJ, JL, and PW) after confirming that all had a central organizing concept, distinct from each other and related to the aim of illustrating the lived experience and influencing factors for why ambulance employees would or would not seek organizational support for mental health. The revision of data ceased when existing themes were being continually re-identified, when all relevant data had been considered, and when the reviewers were satisfied with the thematic map.

### 2.7. Public and Patient Involvement and Engagement (PPIE)

The research protocol was rigorously reviewed by PPIE representatives. Seven representatives were patients or relatives of patients with lived experience of using emergency medical ambulance services. Additionally, a reference group of fifteen EMS employees from clinical and non-clinical roles, considered as PPIE for the purposes of this study, contributed valuable perspectives.

### 2.8. Sensitivity Analysis

The quality assessment of the included studies guided a sensitivity analysis, evaluating the robustness of the synthesis by determining whether excluding low-quality articles influenced the findings and our confidence in them [29,30]. Low-quality articles were identified by calculating a summary score for each paper, by summing the total score obtained across relevant quality checklist items and dividing by the total possible score (i.e., 28 − (number of “n/a” × 2)). For primary articles, we applied Kmet et al. [21]’s cutoff points, removing articles with a summary score below 0.55 to differentiate between higher- and lower-quality reporting. For the gray literature, known for inherently higher bias levels, we set a cutoff score of 0.75. The influence of removing these articles on the findings was assessed by evaluating the following:

- Whether any codes were left without associated references;
- Any changes in the number and meaningfulness of references supporting the codes and assessing whether codes were still supported by the data;
- Any change in the distribution of codes associated with a theme and whether such themes were still representative of the data.

Following the completion of the sensitivity analysis, the reporting of this review was guided by the PRISMA Statement and the RTA Reporting Guideline (RTARG) [19,31].

## 3. Results

### 3.1. Search Results

From a total of 34 included papers, 23 articles [32,33,34,35,36,37,38,39,40,41,42,43,44,45,46,47,48,49,50,51,52,53,54] were identified from database and register searches and 11 articles [55,56,57,58,59,60,61,62,63,64,65] by other methods (see Supplementary Material S5, Figure S1). The articles represented a broad spectrum of study design and grey literature. Published research included 12 qualitative [32,33,34,36,37,38,45,49,51,53,60,63], 8 quantitative [41,43,44,47,50,56,58,61], and 6 mixed-methods studies [48,52,55,62,64,65], alongside 3 systematic reviews [39,54,66], and 1 randomized controlled trial [35]. Grey literature included 2 industry briefings [42,59], 1 quality improvement study [57], and 1 opinion piece [40]. Only one article, Clompus and Albarran’s [51] qualitative study, was forwarded to a third reviewer (KS) due to uncertainty about its relevance, who agreed it warranted inclusion. Data from eight of the included primary studies were also discussed within the three systematic reviews [33,36,37,38,45,51,57,60]. To manage information duplication, data from primary and systematic review studies were independently extracted and integrated during qualitative analysis. This approach strengthened results, as overlapping data encouraged reflexion and consideration of each source’s unique insights [28,67]. 

The included articles were published between 2007 and 2023, originated from 15 countries worldwide, included 20,354 EMS employees (alongside 169 papers included in Williams et al. [52] systematic review, where sample sizes were not reported). Sample sizes ranged from 6 to 4022. A total of n = 30 articles described supportive interventions offered by EMS organizations. Characterized using the TIDieR checklist, the most commonly reported interventions were post-incident debriefing, out-sourced EAP’s, and peer support networks (see Supplementary Material S6, Table S3). The main characteristics of the included studies are presented in Appendix A, Table A2.

### 3.2. Quality Assessment Results

The quality assessment consensus is presented in Supplementary Material S7, Table S4. Kappa statistics of reviewers’ agreement to checklist answers were calculated following binary categorization of answers into 1 = yes or partially reported and 0 = no, answer not reported. By comparing these binary responses, a kappa above 0.90 was determined, which is considered a very good level of agreement [20].

A total of n = 38 checklists were applied to the n = 34 studies (due to two checklists being applied to mixed-methods studies). The majority of studies were determined as low risk for bias (n = 18), with n = 8 studies assessed as unclear and n = 6 studies deemed high risk. Common methodological reporting issues were the lack of reflexivity in qualitative studies, poorly defined outcome measures in quantitative studies, and a lack of objectivity in gray articles.

### 3.3. Summary of Findings

The results were based on 102 codes systematically constructed from 34 studies between May and June 2024. Aided by NVivo software (see Supplementary Material S8, Figure S2), the codes were utilized to construct themes that represented data linked to employee perceptions of support delivery factors and organizational factors influencing the barriers to and enablers of utilization of organizational support for mental health. A number of data from quantitative studies were qualitized; e.g., frequency and percentage data from Ntatamala and Adams’ [47] quantitative survey were transformed into a textual statement of “Over a third of participants reporting barriers to help-seeking for work related stress, feared that services were not confidential, and this factor would be a barrier to utilizing support.”

Once the data had been qualitized, integrated, and thematically analyzed, the identified themes of employee (in)ability to ask for help, tailored person-centered support, and education and training about mental health were overarched by a central theme of organizational culture. This review reinforced existing knowledge that barriers include perceived stigma, fear that disclosure would negatively affect careers, and the importance of being provided with time at work, alongside peer and manager-led support for normalizing help-seeking behavior. We also identified new information that EMS employees perceive support as obligatory, rather than genuine, care from their employer, alongside machismo and the perceived importance of EMS context-specific and person-centered support options. These themes are presented in the context of barriers to and enablers of organizational support. An overview of the constructed themes and associated codes is presented in Supplementary Material S9, Table S5, and illustrative quotes supporting the following themes are provided in Supplementary Material S10, Table S6.

#### 3.3.1. Overarching Theme: Organizational Culture

Thirty-one articles discussed the ways in which employees perceived that EMS culture shaped how they interact, make decisions, and approach their mental health at work. Perceived culture underpinned the other three sub-themes by both hindering support utilization (due to resource limitations and cultural norms) and facilitating support utilization (when aligned with evidence-based interventions and cultural norms). Moreover, EMS employees work within a culture where organizational support was perceived as obligatory rather than genuine, leading to feelings of being expendable and undervalued.

Distressing experiences were frequently overlooked by the organization and were compounded by fear that disclosing mental health issues may result in adverse career implications [64]. Aggression and violence were viewed as simply part of the job, and those who could not accept this did not last long in the profession.

Inconsistent strategic commitment to employee well-being, poor communication, and stigmatizing leadership attitudes fostered an environment where mental health discussions were avoided. Machismo, characterized by an exaggerated sense of masculine pride, often linked to traits like dominance, aggression, and an aversion to showing vulnerability, hindered the acknowledgment of symptoms of mental ill health and help-seeking. In EMS, this reluctance, particularly among males, stemmed from perceived weakness, fear of judgment, and cultural expectations to “tough it out.” The need for belonging, togetherness, and camaraderie was prevalent but intertwined with concepts of “brotherhood” and “bravado”, where expressing emotion was seen as a weakness and even emasculating [54].

Female employees found this masculine culture challenging at times and reported having to adopt “masculine qualities” such as being ”loud, robust, and engaging in male-focused banter” in order to fit in. Furthermore, for those who were able to express their emotions, this was conflated with weakness, seen as a lack of resilience, and an indicator that they “shouldn’t be in the job” [64]. A culture where weakness was conflated with emotional expression was a common feature felt across the whole workforce [64]. When combined with perceptions that the organization may not take mental health seriously, EMS culture itself becomes a powerful barrier to employees disclosing symptoms of mental ill health and utilizing support [38,62]. Furthermore, a lack of an open culture contributed to a lack of empathy, understanding, and resentment between colleagues [33,55]. The importance of prioritizing employee mental health and organizational encouragement of self-care and emotional disclosure, with the delivery of patient care, was highlighted and encapsulated by one paramedic: “How can I offer support [---] when we can’t even take care of ourselves?” [41].

In summary, the everyday working habits of EMS employees were strongly influenced by organizational culture. There appeared to be a misalignment between the needs of employees and EMS culture, especially around communication and normalization of accessing support.

#### 3.3.2. Subtheme 1: Employee (In)Ability to Talk About Mental Health and Ask for Help

Thirty-two articles discussed whether individual employees would disclose feelings and ask for help if needed. We found that organizational structure may complicate the landscape with a pervasive stigma around mental health, discouraging open discourse and perpetuating a culture that values stoicism [66]. Fear of appearing weak when discussing the impact of work on mental health and concerns that self-care might conflict with assigned job responsibilities can deter employees from speaking up and reaching out for help [39].

A lack of time at work was a particular barrier to accessing support. It was perceived that integrating time at work into organizational strategy to enable employees to reflect upon their own experiences and needs would help employees to feel valued and provide opportunity to connect staff with services designed to help [64].

A perceived lack of confidentiality when disclosing symptoms of poor mental health to others within the organization was described [64]. EMS employees operate in a protocol-driven, patient-centered environment. The lack of encouragement, time, and self-care directives undermines confidence in discussing mental health and seeking help. EMS employees feel it is important to feel safe and genuinely heard and to be met with genuine concern and empathy when they ask for help [37].

Employees’ ability to talk about their mental health was influenced by pre-EMS life experiences, such as traumatic childhood events. Such experiences may be a driving altruistic factor as to why some join EMS in the first place but may leave some employees vulnerable to certain types of incidents or general psychological stressors and could contribute to difficulties in seeking support. It was clear that work-related incidents could affect employees differently [51]. Incidents involving children and suicide, especially the suicide of a colleague, were seen as high-risk events for worsening employee mental health, especially if the aftermath was poorly managed by the organization [62].

Posttraumatic stress disorder (PTSD) was a concern, and factors such as avoidant coping were discussed alongside delayed psychological impact following incidents. These factors may create barriers to utilizing support as employees may not be ready or able to discuss their experiences within the early weeks and months following an event, when commonly used interventions such as critical incident stress management (CISM) and trauma risk management (TRiM) interventions are often provided. Consequently, EMS systems should be designed to adapt and respond to individual needs and provide support on a person-centered basis [59].

Speaking with peers was viewed as beneficial due to shared humor and camaraderie, which help to build positive workplace relationships and support systems [49].

However, building trust takes time, and employees may hesitate to access organizational support, including formalized peer support networks, until trust is established. Therefore, those early in their careers may rely on informal support outside the organization [36,37]. In contrast, more experienced employees are less likely to share their feelings and experiences with friends and family as they do not want to burden them [36].

Consequently, longer service can facilitate support use as experienced employees were more likely to utilize organizational support compared with newer recruits, despite reporting that they found it less useful. However, mistrust of organizational processes hinders support utilization for all employees regardless of length of service. This combined with the fear of burdening family and friends may leave employees feeling like they have nowhere to turn. Moreover, experienced employees may feel less comfortable discussing their mental health with managers [34]. Hierarchy seemed to create barriers in both directions, leading to a tendency to withhold and avoid emotional interactions observed across different hierarchical levels [34].

In summary, a perceived lack of confidentiality and encouragement to talk about mental health and well-being, combined with a lack of genuine concern and knowledge about what support is available to help, hampered the ability of EMS employees to discuss their feelings and utilize support when required.

#### 3.3.3. Subtheme 2: Provision and Utilization of Person-Centered Support Tailored to the EMS Context

Thirty-three articles discussed the provision of support offered by EMS organizations. A lack of easily accessible, timely, and useful support; insufficient choice; and a shortage of options tailored to individual needs were identified. These shortcomings discouraged some staff from accessing support due to doubts about effectiveness and fear that support could be actively unhelpful. A lack of information about evidence-based interventions was identified. For example, post-incident debriefing was a commonly discussed concept for enabling EMS employees to talk about their experiences and associated emotion. However, opinions about the usefulness and safety of this approach were mixed; it was perceived as useful for encouraging discussion, while others felt uncomfortable or unprepared to share their experiences so soon after the incident [64]. Formal debriefing practices such as CISM and TRiM were discussed. Both approaches addressed traumatic experiences; CISM focused on immediate post-incident support using debriefing and defusing techniques with the aim of reducing the risk of psychological damage, and TRiM provided immediate and follow-up support, educating employees about trauma responses and providing peer-to-peer individual/group risk assessments, with the aim of spotting signs of distress through a structured process, which might otherwise go unnoticed. Despite evidence that such approaches provide a structured risk assessment, which may be useful for assessing psychological symptoms and helpful for reducing stigma associated with mental health, these concepts were discussed with concern about potential harm and embedding negative feelings that lead to resentment [40]. In contrast, engaging in formal peer support programs for mutual support and emotional expression about an event was viewed in a positive light [54].

An experienced employee reminisced about peer support during their early career, when shifts were equally split between time spent in the vehicle and at the station, which allowed time for training and peer-to-peer debriefing. Time at the station has been eroded over the past 20 years, and newer recruits now only know a constant state of busyness [49].

Organizational culture has not evolved to maintain these supportive units of time for peer-to-peer debriefing and destressing as part of a preventative and proactive strategy. Therefore, formal peer-to-peer interventions and networks set up by EMS organizations that make time for peer-to-peer voice at work were perceived as being important. This shift toward reduced time together at the station has created a system that now relies upon employees to pluck up the courage to speak out, rather than one where taking time to reflect and talk is normalized. Evidence suggests that relying on individuals to ask for help is not working, since employees want to be asked about their well-being [60]. When EMS organizations do not ask whether employees are okay or provide time and supportive interventions at work, employees may not speak up and utilize the available support. Preventative systems that look and listen for all employees, rather than standing by and offering reactive support for those already distressed, should be provided [59].

Accessibility of the support offered was a key factor, with processes to accessing support reported as being onerous and a significant barrier (and in the U.S., prohibitively expensive for some individuals when the organization did not pay for supportive services). The visibility of how support is accessed and stigma were also barriers as some support services required employees to make themselves publicly known to be needing support [53].

The speed at which support was provided was also discussed. EMS employees provide immediate response to emergencies and appeared to hold expectations of a quick response when support was requested for themselves [59]. When responses were not provided quickly, or in some cases not provided at all, this may lead to negative perceptions, feed dissatisfaction, and create barriers for future service utilization [65].

An interesting aspect was the importance of talking with those who understood the unique EMS context. Scenarios were discussed where employee assistance providers were left in tears after hearing the stories of EMS employees, and employees were left feeling unsupported following such interactions or felt their experience could not be fully understood by someone outside the profession [63].

Concern about competency and the additional burden associated with formal peer-to-peer support was discussed, although this was outweighed by the importance of support facilitated and designed by those who understood the context. A call for more mental health professionals familiar with the EMS field was made [64].

Employees wanted a range of useful and evidence-based interventions delivered by trained, confident, and non-judgmental individuals who understood the context. Strengthening employee perceptions and confidence in the effectiveness of the support offered would increase confidence in using services when required, and including cognitive reappraisal within supportive structures was identified as a useful approach for reframing negative narratives [33].

Optional and mandatory organizational mental health support provision for EMS employees following traumatic calls was discussed. Mandatory approaches were perceived as useful for reducing stigma and signaling organizational commitment to employee mental health and well-being. When EMS organizations mandated employees to attend supportive sessions, individuals were not forced to participate if they did not wish to, although the practice was seen as useful for connecting employees perceived to be resistant to supportive offers. Encouraging employees to attend in support of others was a suggested method for including individuals perceived as reluctant to attend for their own benefit [45]. However, evidence suggested that making participation with support mandatory may make it less effective [44]. There was no empirical evidence to support or to refute the effectiveness of mandating time for support in reducing barriers to participation. However, mandating supportive time following certain incidents that were deemed as high-risk, such as critical incidents involving children, may be beneficial.

Overall, the adequacy and timing of the organizational support provided to employees is crucial as evidence suggests that this influences service utilization; mediates employee outcomes; and, in turn, improves patient care: “…the feeling of an abundance or lack of support directly affects the quality of care delivered to the patient” [40].

#### 3.3.4. Subtheme 3: Education and Training

Twenty articles discussed barriers to and enablers of organizational support in the context of training and education. Working for EMS carried with it an assumption that employees, by the nature of their work, would be well trained in understanding mental health, identifying symptoms of poor mental health, and knowing how to seek help when needed. However, the evidence suggested that employees felt underprepared for the psychological challenges of their EMS role due to a lack of training and protocols [53,64].

Reactionary culture was cited as a barrier to knowing when to utilize support due to the lack of time at work for training and education about the recognition of symptoms in themselves and others. Furthermore, employees were not involved in decision-making about intervention development and what support is offered, which fed into previously discussed issues of not knowing what support is available and underconfidence in effectiveness. Active engagement of employees with the development of supportive interventions could reduce the barriers to utilization of services [65].

A paramedic discussed the importance of co-production by sharing their experience of how the fire service involved their employees in developing organizational goals, mission, and values and the benefits this had for creating a cohesive workforce [65]. This reinforced the importance of leadership, of including employees in the creation of education and training, and ensuring that managers are also well trained. The influence of manager support for employees was discussed across the included articles. Managers were often the first point of contact when employees were exhibiting symptoms of poor mental health or were seeking support, and how managers dealt with this interaction was pivotal to employee well-being and support utilization [34].

Providing managers with training that sensitizes them to the effects of stress and enables them to detect stress in others should be an essential part of a manager’s job role. Emotional awareness and preparedness training would ensure all employees are equipped to detect changes in mental health. When this is combined with organizational readiness to provide evidence-based training and education, the barriers for employees seeking help when needed are reduced [39].

Training should aim to build knowledge about and confidence in utilizing support and should include preventative activities such as psychoeducation, burnout prevention, stress management techniques, and healthy coping education. This includes a greater depth of understanding about maladaptive coping such as avoidant-coping and drug and alcohol misuse. Training about suicidal ideation and suicide is also important since despite the known elevated risk among EMS employees, there was a lack of prevention and postvention training and protocols [47]. This links back to a lack of evidence underpinning what EMS organizations should offer their employees, who should offer support, and when. Evaluating the effectiveness of any adopted approach is important, and the evidence suggested that this was not always thought about [64].

The mode of evaluation requires careful consideration. In particular, the limitations of survey methods for examining support service satisfaction among EMS employees were discussed, identifying that appreciation of a service does not always equate to service effectiveness. Furthermore, as reflected in previously discussed themes, employees are often mistrustful of organizational motives and worry that disclosure of feelings may be used against them. This may influence how truthfully employees respond to surveys [64].

Additionally, evaluation methods, such as evaluating services during annual support meetings, may be unhelpful, especially for ambulance stations with less adequately functioning support [45].

In summary, nurturing employees and organizational processes through education and training about recognition, disclosure, and support utilization for mental health were important factors in connecting employees with organizational support.

### 3.4. Sensitivity Analysis Results

Following quality assessment, 6 articles were identified as low-quality based upon the reporting in the included articles, 3 used a quantitative methodology and three were grey articles (3 from the USA, 2 from Canada, and 1 from the UK) [40,42,44,59,61,64]. The codes associated with these articles were removed from the data analysis using NVivo software, leading to a 25% reduction in the underpinning references of the 102 codes. The impact on the themes and underpinning codes were visually assessed by one author (SJ), reviewed and agreed by two others (JL, PW), and recorded in Supplementary Material S11, Table S7.

Themes continued to be supported except for three factors. First, the concepts contained within the subtheme of “employee (in)ability to talk about mental health and ask for help” remained unchanged, despite being weakened by the removal of low-quality studies. The concept of engaging stakeholders to inform decision-making was left unsupported in the overarching “culture” theme. From the “training” theme, the concept of evaluating and measuring effectiveness of the support provided was left unsupported. Finally, the concept of inclusive support that was representative of the workforce, which was nested within the “support provision” theme, was also left unsupported.

These concepts are important, although it could be argued that from an employee lens, concepts such as effectiveness measures and engaging stakeholders may be unfamiliar. The concept of inclusivity is vital to organizational support; nevertheless, the loss of this concept following sensitivity analysis may be attributed to the lack of population representativeness among EMS populations and EMS-related research [68]. Overall, the sensitivity analysis demonstrated that the constructed themes were robust and reflected the voices found in the underlying data.

## 4. Discussion

This systematic review aimed to identify and synthesize what is known about the factors influencing why EMS employees will or will not utilize organizational support for mental health and well-being. We identified 34 articles from the published and gray literature and synthesized the findings to construct one overarching theme and three key subthemes, which align with and expand upon the existing literature. Sensitivity analysis identified that reporting the quality of the included articles did not significantly change the findings.

Perceived barriers included a perception that organizational efforts were obligatory rather than genuine, prevailing machismo, fear of stigma, negative career consequences, and distrust in organizational motives and processes. Additional obstacles included concerns about manager and colleague discretion and ability to support mental health, lack of time at work for support, and insufficient awareness of evidence-based resources and interventions.

Enablers involved belief in organizational values; a culture of self-care, which included time at work for support; proactive measures; and strong leadership commitment. Factors that can be either barriers to or enablers of help-seeking included pre-ambulance life experiences and the impact of work-related incidents, associated coping techniques, and how such experiences shaped attitude and confidence in utilizing support. These factors were identified alongside the quality of communication and support offered [6,60,65].

This review identified that culture lies at the heart of whether employees will or will not utilize support provided at work. Employees want to be proactively asked about their well-being, and if they share their feelings, they want this information to be met with genuine concern, discretion, and evidence-based interventions designed to help. EMS organizations consider employee mental health and well-being through formal norms, such as regulatory obligations and well-being policies. However, informal norms of team dynamics, leadership style, traditions, and rituals appear to be more influential in employee willingness to be open about and seek help for mental health and well-being [69]. This emphasizes the detrimental effects of perceived organizational indifference about employee well-being.

An important factor to consider when discussing organizational support is the variation in operational models across EMS organizations. Differences in staffing structures, service delivery expectations, and workload management can influence employee stress levels, coping mechanisms, and perceptions of organizational support. For instance, high-intensity, resource-limited environments may exacerbate employee distress, while findings from this review suggest that there is appetite for well-supported models with structured mental health initiatives, which may mitigate psychological strain [70].

EMS employees facing mental health challenges encounter several barriers to accessing organizational support regardless of their job role. The perception of organizational obligation, where support is seen as a duty rather than genuine care, can deter help-seeking as employees need genuine psychological safety to share their feelings. Consistent with Caesens et al. [71] and Brunetto et al. [72], feelings of expendability make employees hesitant to seek assistance, perceiving repercussions for doing so or developing perceptions of being deemed replaceable or as lacking aptitude for their role.

Evidence reinforces the importance of individuals taking responsibility for understanding, recognizing, and taking action to look after one’s own mental health and well-being [73]. However, tension between individual and organizational responsibility for well-being is well documented [48]. Therefore, employee-led strategy is insufficient as EMS organizational support policies rely on employees asking for help, despite evidence that self-recognition of mental ill health can be hampered by symptoms [74]. Disclosure of symptoms and the ability to ask for help can also be further compromised by cultural norms.

This is important as the machismo identified in this review may be a contributing factor to creating an environment where misogyny, known to permeate EMS culture, can grow as discussed in a recent review by the English National Freedom to Speak Up Guardian [75]. This report reinforces the view that not only are these concepts barriers to help-seeking, but they may be contributing factors as to why EMS employees need psychological help in the first place and why they are reluctant to utilize organizational support [75,76].

Employees perceived the culture to be overtly “macho” and stigmatizing toward seeking support for mental health, which, in turn, creates barriers to support utilization and aligns with the existing literature [77,78]. Machismo is often associated with male stereotypes that emphasize toughness, self-reliance, and dominance. Such traits may be desirable for coping with the unpredictable nature of emergency work. In EMS contexts, females may also exhibit traits associated with toxic masculinity; Clompus and Albarran [51] found that female paramedics reported adopting masculine qualities by becoming “geezer birds” to fit in.

Beyond individual perceptions, organizational culture plays a pivotal role. A stigmatizing culture can exacerbate barriers to organizational support. Factors such as insufficient time at work and funding for support programs, alongside inconsistent manager support, can further compound help-seeking barriers. Thus, understanding and addressing cultural aspects is essential, not only for ensuring that employees utilize support provided but for enhancing organizational capability, as perceptions of organizational support influence worker behavior and are vital for ensuring that employees feel valued and ready to perform to their best ability [79].

Enablers identified in this review shed light on the benefits of proactive approaches. Employees who believed in organizational goals and values were more likely to seek support. When organizational values aligned with employee well-being, a supportive environment was more likely to be fostered. This aligns with the known literature and resonates with principles of transformational leadership and positive organizational behavior as discussed by Avolio and Gardner [80] and Crawford et al. [81], as well as the EMS organizational briefings included in this review [42,59]. When well-being is prioritized within cultural norms, where preventative measures and stress education are emphasized, employees are more likely to engage with available support systems.

It was clear that genuine leadership commitment to mental health and well-being at work mattered to EMS employees. Leaders who actively supported employee well-being created an environment where seeking help was encouraged. The emphasis on leadership and organizational flexibility aligns with the findings of Neilsen et al. [82], whose systematic review identified that these factors are crucial for fostering a healthy work environment, allowing employees to balance work and personal needs, while reducing stress and enhancing overall health. Despite progress in developing operationally effective managers, EMS organizations still face challenges in cultivating supportive leaders capable of fostering an open culture and connecting employees with necessary support. Prioritizing employee well-being and fostering a supportive culture remains crucial for connecting employees with the support designed to help. This approach emphasizes the importance of organizational support planning that includes person-centered strategies [83]. Person-centered approaches could include connecting employees with experts in specialist services through general practitioners, charitable organizations, or other healthcare avenues.

It appears to be important to employees to be asked how they are (rather than a structured assessment) after a traumatic incident at work to improve support utilization and to ensure people feel valued by their colleagues and employer. Some employees reported difficulties with self-reporting symptoms and felt under-confident and apprehensive about reaching out to colleagues if they perceived them to be experiencing poor mental health.

This may lead to challenges in providing adequate support for employees who may under-report symptoms. Additionally, pre-ambulance adverse life experiences, such as exposure to early childhood traumatic events (often the driving factor for joining EMS), coupled with the cumulative impact of work-related incidents contributed to whether employees would or would not utilize the provided support [51,84,85]. Adverse childhood experiences can increase vulnerability following subsequent traumatic events and lead to more severe anxiety and depression in adulthood [86]. Employees easily triggered by events may experience stigma at work and questions about their suitability for their EMS role, which hampers their ability to speak up and seek help. Conversely, lived experience of adversity may enhance empathy and ability to connect with patients.

The literature emphasizes the value of experiential knowledge for improving care [87]. Those who were perceived as being more resilient due to their lived experiences may be perceived as being less caring or more prone to compassion fatigue [88,89,90]. Previous experiences of support can also inform decision-making about utilizing support, especially if previous experiences were negative or ineffective. Recognizing these complexities and adapting supportive interventions to accommodate differences in factors such as the psychological processing time, severity of post-incident symptoms, and type of support needed allows organizations to tailor support. This, in turn, will help to minimize negative influences of lived experience on clinical decision-making and support EMS organizational goals of reducing unwarranted variation in patient care [91].

The findings of this review underscore the value of trust in facilitating employees to speak about mental health and, in particular, with people who understand their working context. Reports of counselors becoming tearful upon hearing work-related stories and perceived disingenuous understanding of what employees may be experiencing were barriers to engaging with support. Employing experts or training peers who understand the EMS context appears to be a vital link in ensuring that the provided support is meaningful to employees.

### 4.1. Implications

The findings of this review have important implications for public health policy and practice. There is a need for targeted interventions to foster a more supportive and inclusive work environment. Reframing machismo in EMS settings could minimize the negative aspects and help to de-stigmatize help-seeking culture. For example, shifting the narrative from dominance, competition, and toughness to positive aspects of respect, perseverance, and courage could help to transform the culture [92]. This, in turn, could enable employees to seek support when needed.

This review highlights the critical role of leadership in shaping organizational culture and employee well-being. Strong leadership commitment to mental health initiatives, coupled with communication and adequate time and resources, can improve the utilization and perceived effectiveness of support programs. The provision of person-centered support that addresses the diverse needs of employees, including those arising from pre-ambulance life experiences and work-related incidents, may help improve overall mental health outcomes in EMS workforces.

### 4.2. Strengths and Limitations of the Study

The strengths of this review include examination of the gray literature. We also undertook a rigorous methodological approach to strengthen the reliability of the findings. We found the innovative method of integrating qualitative and quantitative data designed by the Joanna Briggs Institute worked well in combining mixed-methods data for analysis. The use of inductive reflexive thematic analysis then ensured that employee voices were captured authentically while minimizing bias. The sensitivity analysis furthered the robustness of results. By removing low-quality studies, we addressed issues of methodological quality and poor reporting while demonstrating the strength of overall conclusions. These methodological choices collectively minimized the common pitfalls associated with mixed-methods systematic reviews [93].

Despite its strengths, the review has limitations. Only one author examined all the literature, and despite a reflexive approach to challenge bias, a single reviewer may inadvertently overlook relevant studies or introduce bias due to individual perspectives or preferences. While this was in part mitigated by recording decision-making and a second reviewer examining 10% of the data, this partial involvement may not fully capture the nuances of all included studies. A more comprehensive dual-reviewer process would enhance reliability and minimize potential oversights. We also note the moderate kappa inter-rater agreement score identified at the article selection process stage, which was likely hampered by the broad eligibility criteria. The criteria were purposefully designed to capture all the available information yet consequently resulted in large volumes of information unrelated to the research question.

The inclusion criteria focus on government/state commissioned EMS ambulance systems may limit generalizability. In some countries, only voluntary or privately funded EMS services may be available, and excluding such services could miss valuable insights. The consideration of broader contexts would enhance external validity and would need to consider the route to support for employees in these services.

Finally, a methodology that includes qualitization of data and reflexive thematic analysis, while valuable for capturing employee voices, can be subjective. Although carefully crafted, the interpretation of these data may vary, potentially affecting the replicability and reliability of findings.

## 5. Conclusions

Barriers to organizational support include prevailing machismo, a perception that support is being offered out of obligation rather than genuine care, cultural stigma about employee mental health, a lack of trust, and inadequate evidence-based resources. Although employees bring their own barriers to the table through lived experience and pre-formed attitudes toward help-seeking, they perceive that barriers to utilizing organizational support are most likely to be reduced by fostering empathetic cultures that prioritize time, money, and resources for employee mental health, while actively encouraging support utilization.

Enablers involved belief in organizational values and a culture of self-care and looking out for others, alongside dedicated time at work for employee mental health and well-being. Furthermore, proactive measures and strong leadership commitment, providing employee awareness and improving employee confidence to speak up, and integrating holistic approaches to organizational support were perceived to enable support utilization.

Factors that can be either barriers or enablers include pre-ambulance life experiences, length of service, the impact of work-related incidents, and the quality of communication and support within the organization. It was also perceived to be important to involve employees and experts in decision-making about what, when, and by whom support should be provided.

Reframing aspects of machismo and providing time at work for proactive (rather than reactive) support for employee mental health and well-being based on a culture of trust and genuine care will likely reduce barriers to support utilization. The future of EMS depends on its workforce, and without cultural normalization of self-care and help-seeking, EMS services risk compromising employee and, in turn, patient outcomes [94].

## Supplementary Materials

The following supporting information can be downloaded at https://www.mdpi.com/article/10.3390/ijerph22040629/s1: Supplementary Material S1: Search Strategy; Supplementary Material S2, Table S1: Webpages searched; Supplementary Material S3, Table S2: Inclusion and exclusion criteria; Supplementary Material S4: Methods—Reflexivity and Theoretical Underpinning; Supplementary Material S5, Figure S1: PRISMA Flow diagram; Supplementary Material S6, Table S3: TIDiER checklist results; Supplementary Material S7, Table S4: Quality assessment of included studies; Supplementary Material S8, Figure S2: Word cloud visual representation; Supplementary Material S9, Table S5: Overview of themes and associated codes; Supplementary Material S10, Table S6: Illustrative quotes supporting identified themes; Supplementary Material S11, Table S7: Assessment of codes following sensitivity analysis.

## Abbreviations

The following abbreviations are used in this manuscript:

EMS	Emergency medical service
EAP	Employee assistance program
TIDieR	Template for Intervention Description and Replication
PRISMA	Preferred Reporting Items for Systematic Review and Meta-Analysis
PICoT	Population, intervention, comparison, outcome, and time
JBI	Joanna Briggs Institute
RTA	Reflexive thematic analysis
PPIE	Public and patient involvement and engagement in research
PTSD	Posttraumatic stress disorder
CISM	Critical incident stress management
TRiM	Trauma risk management

## Appendix A

**Table A1:** Summary of quality appraisal checklist items.

Checklist Type	Question No.	Question
Qualitative [21]	1	Question/objective clearly described?
	2	Design evident and appropriate to answer study question?
	3	Context for the study is clear?
	4	Connection to a theoretical framework/wider body of knowledge?
	5	Sampling strategy described, relevant, and justified?
	6	Data collection methods clearly described and systematic?
	7	Data analysis clearly described, complete, and systematic?
	8	Use of verification procedure(s) to establish credibility of the study?
	9	Conclusions supported by the results?
	10	Reflexivity of the account?
Quantitative [21]	1	Question or objective sufficiently described?
	2	Design evident and appropriate to answer study question?
	3	Method of subject selection (and comparison group selection, if applicable) or source of information/input variables described and appropriate?
	4	Subject (and comparison group, if applicable) characteristics or input variables/information sufficiently described?
	5	If random allocation to treatment group was possible, was it described?
	6	If interventional and blinding of investigators to the intervention was possible, was it reported?
	7	If interventional and blinding of subjects to the intervention was possible, was it reported?
	8	Outcome and (if applicable) exposure measure(s) well defined and robust to measurement/misclassification bias? Means of assessment reported?
	9	Sample size appropriate?
	10	Analysis described and appropriate?
	11	Some estimate of variance is reported for the main results?
	12	Controlled for confounding?
	13	Results reported in sufficient detail?
	14	Do the results support the conclusions?
Systematic review [22]	1	Is the review question clearly and explicitly stated?
	2	Were the inclusion criteria appropriate for the review question?
	3	Was the search strategy appropriate?
	4	Were the sources and resources used to search for studies adequate?
	5	Were the criteria for appraising studies appropriate?
	6	Was critical appraisal conducted by two or more reviewers independently?
	7	Were there methods to minimize errors in data extraction?
	8	Were the methods used to combine studies appropriate?
	9	Was the likelihood of publication bias assessed?
	10	Were recommendations for policy and/or practice supported by the reported data?
	11	Were the specific directives for new research appropriate?
Randomized controlled trial [23]	1	Was true randomization used for assignment of participants to treatment groups?
	2	Was allocation to treatment groups concealed?
	3	Were treatment groups similar at the baseline?
	4	Were participants blind to treatment assignment?
	5	Were those delivering the treatment blind to treatment assignment?
	6	Were treatment groups treated identically other than the intervention of interest?
	7	Were outcome assessors blind to treatment assignment?
	8	Were outcomes measured in the same way for treatment groups?
	9	Were outcomes measured in a reliable way?
	10	Was follow-up complete, and if not, were differences between groups in terms of their follow-up adequately described and analyzed?
	11	Were participants analyzed in the groups to which they were randomized?
	12	Was appropriate statistical analysis used?
	13	Was the trial design appropriate and any deviations from the standard RCT design accounted for in the conduct and analysis of the trial?
Gray literature (AACODS) [24]	1	Authority: Identifying who is responsible for the intellectual content.
	2	Accuracy
	3	Coverage: Are any limits clearly stated?
	4	Objectivity: It is important to identify bias, particularly if it is unstated or unacknowledged.
	5	Date: For the item to inform your research, it needs to have a date that confirms relevance.
	6	Significance: This is a value judgment of the item, in the context of the relevant research area.

**Table A2:** Summary of included studies.

Lead Author/Publication Year/Country	Title/ID	Search Source	Methods	Setting	Population	Sample Size	Phenomenon of Interest	Outcome Measures	Analysis	Results	Strengths	Weaknesses	Citation Search
Adams et al. (2015) [33], Australia	An Interpretative Phenomenological Analysis of Stress and Well-Being in Emergency Medical Dispatchers	Database	Qualitative	Australia–One statewide service with three communication centers	EMDs	n = 16	Stress and well-being of EMDs	Semi-structured interviews	Interpretative phenomological analysis (IPA)	Themes: Operational stress and trauma; organizational stress; post-traumatic growth. Despite their physical distance from the crisis scene, EMDs can experience vicarious trauma through acute and cumulative exposure to traumatic incidents and their perceived lack of control.	In-depth qualitative analysis: Semi-structured interviews with 16 EMDs, allowed for detailed exploration of their experiences. The findings offer practical suggestions for emergency services organizations to mitigate job-related stress and promote better mental health outcomes.	Limited to one statewide service, which may affect the applicability of findings. Lack of reported reflexivity may introduce subjectivity into the findings.	No further relevant studies.
Alzahrani et al. (2017) [57], Saudi Arabia	Improving referral to psychological support unit at Saudi Red Crescent Authority in Riyadh Region	Citation search	Quality improve- ment	Saudi Red Crescent Authority in Riyadh region	EMS employees	n = 17	The study aimed to increase the number of employees referred to the psychological support unit (PSU) by 75% in 2 months.	Several improvement interventions were tested sequentially in three consecutive plan–do–study–act cycles on a weekly basis. PDSA 1: brochures raising awareness of the PSU sent to employee email. PDSA 2: electronic consultation for employees seeking support. PDSA 3: re-engineer referral process to save time and effort and ensure confidentiality.	18-week PDSA cycle. A multidisciplinary team was formed to analyze the problem using quality tools including brainstorming, fishbone diagrams, and flow charts of the PSU processes.	The study revealed that the PSU had low employee utilization due to factors like lack of awareness, privacy concerns, and a lengthy referral process. While the first change idea fell short of the target, the second change had a significant but short-lived impact. The third change, re-engineering the referral process, ultimately proved most effective in increasing referrals to the PSU.	Multidisciplinary approach: The study involved a diverse team from various disciplines, which enriched the analysis and solution development. Practical interventions: The tested change ideas were practical and aimed at real-world improvements in the referral process. The PDSA cycle is well described (p. 3).	The study was extended to 18 weeks after seeing a decline in referrals rather than the expected increase. Changes in referral rates in the context of time of year or any other relevant factors were not discussed. Inconsistent reporting: A lack of information is provided about participant characteristics. Compared with baseline, referral rates appear to stay the same (and in some months, referral rates reduce) on average. Furthermore, the aim in the paper states a 75% target increase, while evidence provided in this article suggests the aim changes to a 50% increase? (One referral increased to 1.5.)	No further relevant studies.
Al-Wathinani et al. (2023) [56], Saudi Arabia	Enhancing Psychological Resilience: Examining the Impact of Managerial Support on Mental Health Outcomes for Saudi Ambulance Personnel	Gray—ProQuest	Quantitative	Western Cape Department of Health situated at 50 EMS bases in Saudi Arabia	EMS personnel employed by the Western Cape Department of Health	n = 354	Examine the importance of managerial support in determining the mental well-being of ambulance personnel in Saudi Arabia.	Cross-sectional survey, with a modified (for local context) questionnaire containing validated scales: Short Warwick–Edinburgh Mental Well-Being Scale to assess mental well-being; a validated nine-item scale—the Managerial Behavior Questionnaire—to assess manager behavior.	Descriptive statistics and generalized linear regression models were used to establish the relationship between managerial support and the mental well-being of EMS personnel.	Manager support was positively associated with the mental well-being of EMS personnel. The findings suggest that focusing on improving organizational management behaviors is a promising strategy for enhancing utilization of mental health interventions among EMS personnel.	Comprehensive analysis: The study provides an in-depth examination of the impact of managerial support on the mental health outcomes of Saudi EMS personnel. Practical implications: Offers valuable insights that could inform policy and managerial practices in EMS.	Survey distribution via social media might introduce selection bias as not all ambulance personnel may be active on these platforms or may choose to participate. This may limit the representativeness of the sample. The study was conducted in Saudi Arabia, and the findings may not be generalizable to EMS personnel in other countries with different cultural, organizational, and healthcare contexts.	Study ID Alzahrani et al. (2017) [57], https://doi.org/10.1136/bmjoq-2017-000089, (accessed on 26 June 2024) added to the included study list.
Auth et al. (2022) [39], UK	Mental health and help seeking among trauma-exposed emergency service staff: a qualitative evidence synthesis	Database	Systematic review	Worldwide	Emergency services workers (ESW) including EMS, police, and fire	n = 671 EMS workers from 17 of the 24 qualitative studies meeting inclusion criteria	Qualitative insights grounded in the perceptions of ESWs, which may help EMS organizations in decision-making about psychological support provision for employees following traumatic incident exposure	Qualitative evidence synthesis	Thematic analysis (inductive) Two overarching constructs: (1) factors contributing to mental health (such as the need for downtime and peer support and reassurance) and (2) factors influencing help-seeking behavior (such as stigma, the content/form/mandatory nature of interventions, and mental health literacy issues including emotional awareness and education).	ESWs are at greater risk of stressor-related psychopathology than the general population. Fourteen descriptive themes emerged from this review, including two overarching constructs: (1) factors contributing to mental health (such as the need for downtime and peer support and reassurance) and (2) factors influencing help-seeking behavior (such as stigma, the content/form/mandatory nature of interventions, and mental health literacy issues including emotional awareness and education). The study concludes that there is a need to optimize workplace support to manage stress and improve health among ESWs. The findings are drawn from the pre-COVID-19 literature; however, core themes are omnirelevant.	The first qualitative evidence synthesis of traumatization and mental health help-seeking in ESWs. The study involved a user advisory group of ambulance management stakeholders and ESWs, which strengthened the design and purpose of the research. A comprehensive search was conducted across four databases from 1980 to March 2020, ensuring a broad scope of the literature.	Only one author screened the database searches, which may introduce bias that could have been mitigated with a dual-screening approach. The study’s data may face challenges in interpretation subjectivity and limited representativeness due to unaddressed differences in organizational structure, culture, and support availability across emergency service organizations.	No further relevant studies included. Interventions for the prevention and management of occupational stress injury in first responders: a rapid overview of reviews, https://doi.org/10.1186/s13643-020-01367-w, (accessed on 26 June 2024) was read and excluded as the population was not EMS (police, fire, and correction officers).
Barber et al. (2015) [44], USA	Survey Reveals Alarming Rates of EMS Provider Stress and Thoughts of Suicide	Database	Quantitative	All 50 US states	EMS employees	n = 4022;	Suicide prevalence and employee support	Cross-sectional survey containing unvalidated scales	Descriptive statistics	More than 1 in 20 EMS providers had attempted suicide (10 times higher than the general population). More than 1 in 6 EMS providers had contemplated suicide, (3 times higher than the general population). The most common factors associated with critical stress among EMS providers were lack of sleep, poor diet, and low pay. The most effective ways to reduce critical stress among EMS providers were talking to peers, family, or friends; exercising; and seeking professional help. The study concluded that EMS providers face a high risk of suicide and critical stress and that there is a need for more awareness, education, and support for their mental health and well-being.	Addresses a critical issue within the EMS community, providing insights into the mental health challenges faced by EMS providers. The survey helped raise awareness about the high levels of stress and the prevalence of suicidal thoughts among EMS providers. The researchers demonstrated that EMS staff were willing to complete a survey about this subject.	No sample size calculation undertaken; therefore, unable to estimate a response rate and probability of error. Unvalidated survey questions–created through expert opinion. Survey was re-shared by recipients via social media, allowing the survey to spread to providers and organizations across the country. Therefore, it is difficult to prove that all participants are from the target population, hampering validity and generalizability.	No further relevant studies.
Canadian Standards Association Group (2018) [42]	Psychological health and safety in the paramedic service organization	Database	National briefing	Canada paramedic services	EMS employees	Summary of evidence, EMS Canada	Voluntary standard of practice for employee well-being	Voluntary standard for Canada ambulance organizations: psychological health and safety management system; management review and continual improvement	Descriptive narrative of evidence	Provides paramedic service organizations and other key stakeholders with requirements and guidance on good practice for the identification and assessment of hazards and management of psychological health and safety (PHS) risks. The key points were as follows: The standard promotes the maintenance of good mental health and addresses illness as well. It recognizes a positive duty for both the individual and the organization. It offers sector-specific guidance for developing and maintaining a psychologically healthy and safe workplace. The standard was commissioned by the Paramedic Association of Canada and developed by CSA Group with funding from Ontario’s Occupational Health, Safety and Prevention Innovation Program. It is important to note that this standard is voluntary.	This paper provides a detailed framework based upon research, expert opinion, and lived experience for managing EMS psychological health and safety, addressing various aspects like commitment, leadership, planning, and implementation. Practical guidance: Offers practical steps and measures for paramedic service organizations to protect and promote psychological health and safety. Considers the unique needs of diverse populations within the organization and emphasizes the importance of soliciting input from these groups.	The success of the standard’s application to everyday practice depends on individual organizations’ resources and commitment, which can vary widely. This paper is specific to paramedic service organizations in Canada and may not be applicable to other contexts or countries.	No further relevant studies.
Carvello et al. (2019) [60], Italy	Peer-support: a coping strategy for nurses working at the Emergency Ambulance Service	Gray—MedNar	Qualitative	One EMS service in north Italy	Emergency ambulance nurses	n = 14	Experiences, opinions, and feelings of EMS nursing staff in relation to the use of the peer supporting model	Semi-structured interviews	Unspecified other than “The interviews were conducted and analyzed by all the researchers after having been faithfully transcribed on digital text documents, reporting in brackets some relevant non-verbal gestures, and after having evaluated the nodes and relationships generated by the NVivo qualitative research software”.	Three themes: (1) the need to receive emotional support from colleagues, (2) individual coping strategies implemented, and (3) participants’ perceptions of peer-supporting as a resource that could facilitate processing personal experiences and feelings concerning subjectively complex events on an emotional level.	Real-world relevance: The paper addresses the significant emotional impact of emergency medical situations on ambulance nurses and the importance of support systems.	The lack of clarity and information about analysis methods, reflexivity, and bias reduction reduces replicability and opportunity to assess the robustness of findings.	No further relevant studies.
Clompus and Albarran (2016) [51], UK	Exploring the nature of resilience in paramedic practice: A psycho-social study	Database	Qualitative	One regional urban and rural paramedic center in England	EMS employees	n = 7	Investigate how paramedics address work challenges and how they become resilient.	Semi-structured interviews	Free association narrative interviewing (FANI)	Four themes: (1) operational stress and trauma, (2) organizational stress, (3) posttraumatic growth, and (4) coping mechanisms. Uniquely, this study uncovered how detachment is used to manage emotions and concluded that there is a need for services to support the emotional needs of paramedics.	Utilizes a psycho-social approach useful for examining the interplay between psychological factors and social support in developing resilience.	With a limited sample size of seven participants (five of which were female) from a single regional center, the study’s findings may not be representative of all paramedics or regions. Examples of interview questions were not provided, reducing the opportunity for replicability. No reflexivity and insight reported to help the reader understand the researcher perspective.	No further relevant studies.
Coyte et al. (2024) [54], Australia	Resilience, posttraumatic growth (PTG) and psychological well-being of paramedicine clinicians	Database	Systematic review	Worldwide	EMS employees	n = 5114 from 13 included studies	Examine and synthesize what is known regarding the PTG, resilience, and psychological well-being of paramedicine clinicians, including the experience of these phenomena and the relationship between them.	Qualitative evidence synthesis: The constant comparison method was used with findings reported using a narrative synthesis.	PTG: Posttraumatic growth inventory. Resilience: Resilience scale (n = 4) (RS-11, RS-14, RS-25). Brief resilience scale (n = 2). Resiliency assessment scale. Psychological well-being: Adapted survey previously used by the World Health Organization regarding healthcare worker well-being. Short Warwick–Edinburgh Mental Well-Being Scale.	Moderate PTG and psychological well-being, with PTG positively linked to active coping and negatively to dysfunctional coping. PTG scores were lower for those with longer shifts and for paramedics compared with EMTs. Resilience levels varied from average to above average, with higher scores for part-time workers. A stoic and masculine workplace culture negatively impacted resilience and emotional expression. Psychological well-being was moderate overall and influenced by management and clinical supervision styles. The study emphasizes the importance of supportive work environments and effective coping strategies.	Recommendations for organizations to support PTG and resilience were provided, emphasizing workplace culture and employment flexibility based on factors identified from this review that were important for employee PTG, resilience, and psychological well-being.	A limited sample size of thirteen articles may not fully represent the diversity of paramedicine contexts and hamper generalizability. Publication bias: The study relied on the published literature, potentially excluding useful unpublished or gray literature. Two authors independently appraised the quality of the included studies; however, the number of authors who selected the included studies, then extracted and analyzed the data, is not reported and may introduce bias and hamper replicability.	No further relevant studies.
Fischer and MacPhee (2017) [55], Canada	Canadian paramedic health and wellness project: Workforce profile and health and wellness trends	Gray—website	Mixed methods	All Canadian EMS organizations	All Canadian paramedics	Phase 1 (survey): n = 2557. Phase 2: 4× focus groups; n = 19 and 1:1 interview n = 10. Phase 3 (physical fitness appraisal): n = 20	Collect baseline information to describe workforce trends, sociodemographic, and current health and wellness of paramedics in Canada.	Phase 1: Canadian Paramedic Health and Wellness Survey (CPHWS), available in both official languages (English and French) and available in electronic and paper formats to paramedics across Canada. Phase 2: In-person focus groups and personal interviews were conducted with paramedics in Nova Scotia, Ontario, Alberta, and British Columbia. One researcher conducted all the interviews, which were audio recorded. Phase 3: Individual physical fitness assessments were conducted with paramedics in Nova Scotia, Ontario, and Alberta. The assessments measured body fitness, bi-manual dexterity, and coordination.	Quantitative: Descriptive (Excel) and inferential statistics (IBM SPSS). Qualitative: Constant comparative method.	Survey and focus group findings indicated a decline in mental well-being where operational factors, organizational climate factors, and barriers to good mental health were identified as pressing challenges. Survey and physical fitness appraisal results identified injury, fatigue, and burnout but suggested that musculoskeletal health and fitness were in line with normative data.	Collaborative approach: The project was supported by multiple organizations, including the Canadian Safety and Security Program (CSSP), Defence Research and Development Canada’s Centre for Security Science (DRDC CSS), the County of Frontenac, the Paramedic Association of Canada (PAC), Paramedic Chiefs of Canada (PCC), and Canadian Union of Public Employees (CUPE). This collaborative approach ensured a wide range of perspectives and expertise were included in the project.	Limited scope: The project was limited to paramedics in Canada and may not be representative of paramedics in other countries. Cross-sectional design: The project used a cross-sectional design, which provides a snapshot of the workforce at a specific point in time. This design does not allow for the examination of changes over time.	No further relevant studies.
Gallagher and McGilloway (2007) [38], Ireland	Living in critical times: The impact of critical incidents on frontline ambulance personnel: a qualitative perspective	Database	Qualitative	One large ambulance service in Ireland	EMS employees	n = 27	Attitudes toward current support services being provided for Irish ambulance service staff who have experienced difficulties related to critical incident stress (CIS).	Semi-structured interviews	Thematic analysis	Low support service uptake due to fears relating to confidentiality and machismo, a perceived lack of concern and support from management, and a need for routinely available professional counseling and stress awareness training were identified.	The study provides rich, detailed accounts of the experiences of EMS personnel.	The study was limited to an all-male sample of EMS employees in Ireland, which may not be representative of paramedics in other countries.	No further relevant studies.
Gouweloos-Trines et al. (2017) [41], multiple	Perceived support at work after critical incidents and its relation to psychological distress: a survey among prehospital providers	Database	Quantitative	Australia, Austria, Canada, Germany, New Zealand, Switzerland, UK, and USA	Paramedics and EMTs	n = 813	Investigate (1) to what extent prehospital providers experience support at work, (2) whether support at work is directly associated with lower distress, and (3) whether availability of a formal peer support system is related to lower distress via perceived colleague support.	Cross-sectional survey: Kessler 10 Psychological Distress Scale; Job Content Questionnaire scales for supervisor support and co-worker support.	Descriptive, inferential statistics, and linear regression. Survey Monkey IBM SPSS	Of the 813 respondents, more than half (56.2%) reported moderate to high risk of psychological distress. Participants did not consistently report feeling supported at work (e.g., 39.4% were not aware of formal peer support). Perceived management support, having enough time to recover after critical incidents, and perceived colleague support were related to lower distress. Availability of formal peer support was indirectly related to lower distress via increased perceived colleague support. The study concluded that prehospital providers at risk of psychological distress may benefit from support from colleagues and management and from having time to recover after critical incidents. Formal peer support may assist providers by increasing their sense of support from colleagues. These findings need to be verified in a longitudinal design.	The study surveyed prehospital providers from eight western industrialized countries, providing a broad perspective.	As a cross-sectional study, causality cannot be established, only associations. The additional question set about caring for sick or injured children in the survey could have introduced selection bias with regards to participation, with those more interested in psychosocial care for injured children being more likely to participate. An indication of the total population size in the included countries is not reported, neither is a sample size power calculation. Therefore, we are unable to determine the representativeness or potential for generalizability of the results.	No further relevant studies. Associations between organizational and incident factors and emotional distress in emergency ambulance personnel, https://doi.org/10.1348/014466505X29639, (accessed on 22 January 2024) was read and excluded as the barriers to and/or facilitators of support were not discussed.
Hadas (2019) [65], USA	Affects of Mental Health Limitations, Leadership Interactions, and Generational Diversity on the Morale of Paramedics in Public and Private Emergency Medical Services	Gray—ProQuest	Mixed methods	Texas, USA	EMS employees	n = 16	Close a gap in the lack of existing knowledge regarding the possible influence of trust in leadership on the job satisfaction and increased morale of employee.	Survey, observation, and interviews	Exploratory qualitative case study, using an inductive analysis model. Coding and triangulation were used to identify themes.	Morale among paramedics in public and private EMS was low, influenced by mental health limitations, leadership interactions, and generational diversity. The level of mental health limitations was high, and they were associated with stress, burnout, compassion fatigue, and PTSD. Leadership interactions were poor, characterized by lack of communication, support, recognition, and feedback. Generational diversity was moderate, reflecting different values, expectations, and preferences among age groups. The study concluded that EMS senior leaders should adopt a positive leadership style, provide mental health resources, and understand generational diversity to improve paramedics’ morale and well-being.	Mixed methods that utilize surveys, observations, and semi-structured interviews, providing in-depth insights into the experiences of EMS employees.	Despite the mixed methods approach, how each study phase influenced or related to the other is unclear; the integration of methods appears to be unclear, unstructured, and unjustified. Relevant baseline/demographic information clearly characterizing the participants is not provided (only “time in service” and “job area” are provided in Table 1, p. 57). No justification of survey size and whether the 16 responses provided a reasonable representation of the research aim.	
Halpern et al. (2008) [36], Canada	Interventions for critical incident stress in emergency medical services: A qualitative study	Database	Qualitative	Toronto, Canada: one large urban ambulance service	EMS ambulance supervisors and frontline workers	n = 60	Explore and describe emergency medical technicians’ (EMTs’) experiences of critical incidents and views about potential interventions, in order to facilitate development of interventions that take into account EMS culture.	1:1 interviews and focus groups. n = 29: 1:1 interviews; n = 31: 8 focus groups.	Qualitative exploratory method using ethnographic content analysis with identical guidelines for both 1:1 interview and focus groups.	Participants identified two workplace resources as important to their recovery in the immediate aftermath of an incident: (1) supervisor support and (2) a brief timeout period in which to talk informally, often with peers. Barriers to accessing such support including difficulties recognizing and acknowledging CIS on the part of both EMTs and supervisors; role restriction and inadequate training in support for supervisors; practical issues such as time pressure; and most pervasively, the culture of stigma within the organization were highlighted.	A comprehensive sample size provided a broad range of perspectives. Purposive sampling that included gender and all job role levels ensured a representative sample. Honorarium provided to thank each participant for their time.	Self-selected participants reported considerable experience with, and concern about, critical incidents, which may reflect the experience of the majority of EMS employees or may suggest that only those with lived experience participate in this type of research. This may introduce bias into the results and hamper the representativeness of findings.	
Halpern et al. (2009) [37], Canada	What makes an incident critical for ambulance workers? Emotional outcomes and implications for intervention	Database	Qualitative	Toronto, Canada: one large urban ambulance service	EMS ambulance supervisors and frontline workers	n = 60	Characterize critical incidents and the emotional responses evoked by them, using a narrative approach.	1:1 interviews and focus groups. n = 29: 1:1 interviews; n = 31: 8 focus groups.	Qualitative exploratory method using ethnographic content analysis with identical guidelines for both 1:1 interviews and focus groups.	EMS employees suffered considerable distress from critical incidents and would welcome interventions. Incidents that were identified as critical commonly involved patient death, often combined with poignancy. These events appeared to evoke vulnerable feelings of inability to help and intense compassion, which led to further emotional, cognitive, and behavioral responses. Difficulty in acknowledging distress and fear of stigma presented significant barriers to accessing support. These barriers may be overcome by educating both ambulance personnel and their supervisors to recognize and tolerate the vulnerable feelings often evoked by critical incidents. While gender and length of service did not seem to impact on evoked emotions, recent recruits may be more open to this type of education. The study found that ambulance workers suffered considerable distress from critical incidents and would welcome interventions.	With interviews from 60 ambulance-based workers, both frontline and supervisors, the study captures a diverse range of perspectives.	The data analysis process is well described (p. 176); however, insufficient information (such as participant ID numbers) is provided to allow the reader to judge whether the interpretation offered in the results section is adequately supported by the data. Data collection procedures are not clearly described; difficult to determine if replicable. No semi-structured/topic interview guide in Supplementary Information.	No further relevant studies.
Hugelius et al. (2014) [45], Sweden	Swedish Ambulance Managers’ Descriptions of Crisis Support for Ambulance Staff After Potentially Traumatic Events	Database	Qualitative	Sweden	Ambulance managers	n = 6	Explore ambulance managers’ experiences of crisis support for ambulance staff following potentially traumatic events (PTEs).	Semi-structured interviews	Content analysis	Five categories were identified: (1) description of a PTE, (2) description and performance of crisis support interventions, (3) impact of working in potentially traumatic situations, (4) the ambulance managers’ role in crisis support interventions, and (5) the ambulance managers’ suggestions for improvement. Ambulance managers described crisis support interventions after a PTE as a single, mandatory group meeting with a structure reminiscent of debriefing. The ambulance managers also expressed doubts about the present structures for crisis support and mentioned an alternative approach, which is more in line with present evidence-based recommendations.	The study underscores the critical role of ambulance managers in providing effective crisis support, highlighting their influence on staff well-being.	Information power or data saturation was not discussed in the context of this small sample. Conclusions were only partially supported by the data—the quotes provided do not always support the conclusion that the study shows an overall strong desire on the part of the ambulance managers to protect and support ambulance personnel who are exposed to work-related PTEs. Some ambulance managers expressed a fear of “overdoing” the supportive approach and of overdramatizing the reactions among the ambulance personnel, which could lead to non-supportive interventions. The data stated that some managers thought that the issue was a high priority, and others reported finding it difficult to find time and money to perform crisis support interventions.	No further relevant studies.
Jackson and Romano (2017) [40], UK	Optimizing Workplace Support to Manage Stress and Improve Health	Database	Opinion piece	UK	Comment on UK ambulance services	n/a	Workplace support for employee mental health and stress.	Organizational support; coping methods; peer-to-peer screening	Narrative summary of the literature and opinion	EMS personnel experience high stress from unpredictable incidents, managerial pressure, long hours, and public scrutiny, leading to mental health issues and poor quality of life, with inadequate management support and ineffective debriefing methods with little or no evidence base, although TRiM is suggested as a helpful peer-to-peer screening tool.	The article is relevant to the topic of workplace stress management in emergency services, offering an insight into the issues surrounding workplace and manager support.	This viewpoint article may lack objectivity, and any reflection about bias is not discussed by the authors.	No further relevant studies.
Kellner et al. (2019) [34], Australia	Barriers to the Frontline Manager Support for high-trauma workers, https://doi.org/10.1108/PR-10-2018-0397 (accessed on 22 February 2024).	Database	Qualitative	Three Australian ambulance service organizations	Emergency dispatch officers, patient transport officers, paramedics, frontline managers, middle management, upper management, leadership, and union representatives	n = 72	Identify and understand the barriers to provision of different types of support and how these barriers affect support quality or quantity.	Semi-structured convergent interviewing	Inductive coding and categorizing	Nine barriers were identified that prevent frontline managers (FLMs) from providing optimal support to employees in high-trauma workplaces, such as ambulance services. Three overarching categories: 1. Frontline manager barriers ((1) training availability, (2) attitude and empathy and (3) mental health); 2. Workplace barriers ((4) physical proximity, (5) time restrictions, and (6) workload restraints); 3. Employee barriers ((7) status differences, (8) relationship integrity, and (9) attitude). The authors proposed a model of the barriers to optimal employee support.	This paper provides a comprehensive model of barriers to optimal employee support by juxtaposing House’s (1981) support framework with the study findings. It contributes to a reconceptualization of the relationship between employees and direct managers, which is particularly relevant in high-trauma contexts. Random cross-checking of a selection of coded data by team members enhanced the internal validity of the results.	Lack of justification of methods: The authors state that no further codes were developed and data saturation was reached after coding 20 interviews. No justification is given for the high number of interviews undertaken (n = 72) or explanation as to whether the data for all interviews were examined or stopped when data saturation was determined. The results of 1216 telephone surveys were mentioned but not reported as the authors state that the qualitative interviews were most suited to answering the research question. What happened to these data and whether these data influenced or related to the qualitative interviews are not discussed.	No further relevant studies.
Kling (2020) [58], USA	Needs Assessment for Mental Health Support Towards Emergency Medical Service (EMS) Personnel	ProQuest	Quantitative	Tidewater EMS	Paramedics and firefighters with a minimum qualification of EMT-Basic	n = 153	Occupational needs of EMS providers to determine if they are receiving resources within their organization to cope with occupational stressors.	Cross-sectional survey including validated scales: Professional Quality of Life Scale (ProQoL), Brief Resilience Scale (BRS), Interpersonal Support Evaluation List-12 (ISEL-12), Brief RCOPE, and Occupational Needs survey.	Descriptive statistics and correlational analysis	The results revealed that burnout and secondary traumatic stress were important factors for determining occupational turnover among EMS personnel. Furthermore, EMS providers reported occupational needs such as easier access to mental health, improved staff relations, adequate staffing, and improved shift hours were needed within their organization.	Providing the mean and standard deviation of correlations informs understanding about the distribution and variability of the data, providing more context to the correlation. Utilizing well-established and validated tools enhances the accuracy and reliability of the collected data, which strengthens the overall credibility of the research findings.	The questionnaire was lengthy, with over 70 items across five instruments, leading to some participants skipping sections or not completing the survey fully, resulting in incomplete data. Self-reported surveys can introduce bias as participants might not accurately report their experiences or feelings. Follow-up invitation emails and incentives are recommended in future studies to counteract the low response rate	
Lawn et al. (2020) [66], Australia	The effects of emergency medical service work on the psychological, physical, and social well-being of ambulance personnel: a systematic review of qualitative research	Database	Systematic review	1 January 2000 to October 2018: Ovid Medline database, and incorporated both subject headings (MeSH: Medical subject headings) and text words, and then translated into the PsycInfo, Ovid EMcare, CINAHL, and Scopus databases	Paramedics; emergency first responders (focus on paramedics)	n = 2514 EMS workers from 25 of the 39 studies where sample sizes were reported	Lived experience of ambulance personnel to identify the gaps in the provision of support and care and the challenges faced by those who experience psychological distress as a consequence of their everyday work.	Qualitative evidence synthesis	Thematic narrative synthesis	Several factors present in the day-to-day work of ambulance personnel and in how organizational management acknowledges and responds were identified as being significant and contributing to mental health and well-being or increasing the risk for developing conditions such as PTSD, depression, and anxiety. Ambulance personnel articulated their well-being needs across four key areas: organizational support, informal support, use of humor, and individual mechanisms to cope such as detachment and external supports.	This study systematically reviewed qualitative research published from 2000 to 2018, providing a broad perspective on the topic.	The review is limited to peer-reviewed qualitative research, which may exclude relevant quantitative studies or the gray literature.	No further relevant studies. Lewis-Schroeder et al. (2018) Conceptualization, assessment, and treatment of traumatic stress in first responders: a review of critical issues, https://doi.org/10.1097/HRP.0000000000000176, (accessed on 28 June 2024) was read and excluded as the sample was <50% prehospital EMS. Occupational and organizational Issues in Emergency Medical Services Behavioural Health, DOI: https://doi.org/10.1080/15555240802243120, (accessed on 29 June 2024) was read and excluded as the barriers to and/or facilitators of support were not discussed.
Lilly et al. (2019) [35], USA and Canada	Destress 9-1-1—an online mindfulness-based intervention in reducing stress among emergency medical dispatchers: a randomized controlled trial	Database	Randomized controlled trial	USA and Canada 911 call centers	Active-duty 911 emergency medical dispatchers (EMD) from the USA and Canada randomized and assigned to an intervention or wait list control condition	n = 323	A randomized controlled trial to test the efficacy of a 7-week online mindfulness-based intervention (MBI) tailored to the EMD workforce	Calgary Symptoms of Stress Inventory (C-SOSI); the Mindful Attention Awareness Scale (MAAS)	Repeated measures mixed effects models, with differences assessed by interaction terms between randomization groups and time points.	The study found that over half of the respondents were at moderate to high risk of psychological distress. The intervention group showed significant reductions in stress compared with the control group, both post-intervention and at a 3-month follow-up. While mindfulness scores did not differ between groups, increased mindfulness was linked to greater stress reduction for all participants. The study concluded that a short, weekly online mindfulness-based intervention (MBI) for emergency medical dispatchers effectively reduced stress, suggesting that tailored online MBIs could be beneficial for employees in challenging work environments.	The study developed a 7-week online mindfulness-based intervention (MBI) specifically designed for emergency medical dispatchers (EMDs). This customization acknowledges the unique stressors faced by EMDs. The authors undertook a sensitivity analysis to determine the level of difference in response between completers and non-completers needed to change the analysis results at time point 2 using multiple imputation. This analysis suggested that the results were robust to the impact of bias introduced by differential attrition across groups (attrition was higher among control participants).	The study did not find a significant difference in mindfulness scores between the intervention and control groups. This suggests that the mindfulness component alone may not account for stress reduction. Participants recruited to complete the baseline survey on a rolling basis over an 18-month period may introduce bias and cross-contamination effects. The number of call centers enrolled is not reported, neither is the proportion of EMDs from each call center; potential confounding factors and influence of cross-contamination are not discussed.	No further relevant studies.
Loudoun et al. (2020) [49], Australia	The role of peer-to-peer voice in severe work environments: organizational facilitators and barriers	Database	Qualitative	One Australian state EMS	Paramedics	n = 28 (10 in manager roles)	Paramedics and the formal and informal voice mechanisms used to safeguard their well-being	Semi-structured interviews	Thematic content analysis, NVivo	Three themes: accessing workplace support, call-out processes and classification, and implications of work changes to access informal peer-to-peer voice. Losing peer-to-peer voice can lead to the build-up of stress that could otherwise be mitigated, resulting in diminished well-being.	Theoretical foundation utilizing conservation of resources theory to frame the study analysis provided a solid theoretical basis for the research.	Information about how the semi-structured interview guide was developed or a copy of the guide was not provided. It is unclear who facilitated the interviews: Three researchers performed the interviews together or separately? Limited information about how thematic content analysis was undertaken hampers replicability.	No further relevant studies.
Mackinnon et al. (2020) [43], Australia	Risk of psychological distress, pervasiveness of stigma and utilization of support services: Exploring paramedic perceptions, https://doi.org/10.33151/ajp.17.764 (accessed on 2 January 2024).	Database	Quantitative	Australia and New Zealand	86% paramedics/14% ambulance volunteers	n = 184	Availability of, barriers to, and facilitators of uptake of care and mental health support	Cross-sectional survey: Kessler 10 Psychological Distress Scale; perceptions of access and stigma surrounding organizational MH support services	Descriptive and inferential statistics: Qualtrics/SPSS: Between-group comparisons were made between continuous and categorical variables through a series of independent samples *t*-tests and one-way ANOVA analyses, and between categorical variables via a series of chi-squares. Open-ended responses to questions were coded into categorical themes.	Paramedics are at a greater risk of psychological distress than the general population. Of the participants, 27% reported experiencing high or very high levels of psychological distress. Those who had encountered at least one adverse event at work had higher distress scores compared with those who had not. Additionally, over half of the participants felt there was stigma associated with seeking mental health support from both paramedic colleagues (51%) and managerial staff (54%).	Use of the well-validated Kessler Psychological Distress Scale enhanced the validity of findings.	Convenience and snowball sampling utilized for participant recruitment combined with voluntary self-selection may have led to recruitment bias.	No further relevant studies.
National EMS Management Association (USA) (2016) [64]	National Survey on EMS Mental Health Services	Website: National EMS Management Association (USA)	Quantitative	All 50 US states	EMTs, paramedics, EMS managers, and medical directors	n = 2200	Provide a snapshot of the resources, program, and services EMS agencies provide to EMS practitioners to help them cope with the stress of the job, to maintain their mental health and well-being, and to seek help when they need it.	Cross-sectional survey designed by expert opinion with no validated scales	Descriptive statistics	The survey identified that EMS mental health services are reported as inadequate, and a more comprehensive and accessible mental health services for EMS practitioners is needed. The majority of participants were dissatisfied with the MH services provided by their employers, while nearly half believe that their EMS agency does not consider the mental health and well-being a priority. Of the EMS agencies, 37% provided no MH support, and 42% provided no health and wellness services.	The paper plays a role in raising awareness about the mental health needs of EMS practitioners and advocating for better support systems.	Data collection items and analysis steps are not clearly described. The survey had a relatively low response rate, with only 2200 responses out of the total number of EMS practitioners in the United States.	No further relevant studies.
Ntatamala et al. (2022) [47], South Africa	The Correlates of Post-Traumatic Stress Disorder in Ambulance Personnel and Barriers Faced in Accessing Care for Work-Related Stress	Database	Quantitative	Western Cape Department of Health	Ambulance personnel situated at 50 ambulance bases	n = 388	Factors associated with increased risk for PTSD in ambulance personnel and the barriers faced in accessing support for work-related stress	Cross-sectional survey: Author questionnaire (sociodemographic data), CAGE questionnaire (problem drinking), and questions on barriers faced in seeking help for work-related stress and preferred sources of support; impact of Event Scale-Revised (IES-R); Connor-Davidson Resilience Scale (CD-RISC); EMS Critical Incident Inventory (CII); EMS Chronic Stress Questionnaire (EMS-CSQ); SF-36 Quality of Life Questionnaire (SF-36 QOL)	Descriptive and inferential statistics, Stata 14.0	A 30% prevalence of PTSD, with higher risk linked to smoking, illicit drug use, problem drinking, mental health conditions, medical treatments, critical incident stress, and chronic work-related stress (WRS), was identified. Of the participants, 55% were female, with a median age of 38 (IQR 31–44). Barriers to seeking help included concerns about confidentiality and career impact. The findings highlight the need for workplace interventions to address WRS and to improve support for ambulance personnel.	A range of validated scales provided a robust and multi-faceted approach to data collection.	Ambulance personnel who chose not to participate may be different from those who did, which may alter the overall PTSD prevalence obtained; selection bias. Social desirability bias may have occurred when participants responded in a socially acceptable manner (e.g., underreporting mental health symptoms and substance use), which could have led to reduced study estimates. Recall bias may also have arisen as the questionnaire required participants to rate symptoms based on a traumatic event that was experienced up to 6 months earlier.	No further relevant studies.
Paramedic Chiefs of Canada (2014) [59]	Operational Stress Injury in Paramedic Services: A Briefing to the Paramedic Chiefs of Canada	Website: Paramedic Association of Canada	Committee briefing	Canada paramedic services	EMS	N/A	Overview of employee support options	Evidence summary by an ad hoc committee of experts	Descriptive narrative of evidence	Summary of evidence and suggested action: context, needs, programs, and services; intervention and prevention; GHQ-12; CISM core components	This article thoroughly examines the causes, effects, and management strategies for occupational stress injury, offering a holistic view of the issue and suggesting shared responsibility among employees, government, unions, and other stakeholders.	A lack of a systematic approach to identifying the relevant evidence may introduce bias to the narrative and recommendations.	No further relevant studies.
Phung et al. (2022) [48], UK	The experiences and perceptions of well-being provision among English ambulance services staff: a multi-method qualitative study	Database	Mixed methods	English EMS organizations	Health and well-being leads, ambulance and control room staff	n = 8 health and well-being leads in eight trusts, as well as n = 25 ambulance and control room staff across three trusts	Understand how ambulance service trusts in England deal with staff health and well-being, as well as how employees perceive and use well-being services.	Semi-structured telephone interviews and documentary analysis of ambulance trust policies on well-being	Framework analysis using NVivo for qualitative interviews. Directed-content documentary analysis of staff well-being policies.	Ambulance service work can impact upon physical and mental health, which necessitates effective support for staff mental health and well-being. Line managers should be trained to identify and implement support for their staff. The effectiveness of the support depends also, partly, on the nature of the organizational culture, which is primarily set by those managers that staff interact directly with alongside the availability, range, and accessibility of any offers.	The analysis of n = 57 employee well-being policy documents across English EMS trusts contributes to a comprehensive understanding of the organizational approaches to staff well-being.	This investigation was conducted both before and during the UK’s first COVID-19 pandemic wave; therefore, the results may be influenced by the unique circumstances of that period. The lack of reported reflexivity and member checking/verification may introduce bias and hamper the representativeness of the findings.	No further relevant studies.
Powell et al. (2023), [53] UK	A qualitative analysis of stressors affecting 999 ambulance call handlers’ mental health and well-being	Database	Qualitative	One UK NHS ambulance organization	Full-time call handlers who operate the 999-EMS call service	n = 18	Understand how 999 EMS call handlers experience job stressors that may lead to burnout and how these stressors could be reduced based on modification of job and personal factors	Semi-structured 1:1 telephone interviews	Thematic analysis using a preliminary coding frame inductively created from interviews on sources of burnout, and personal mechanisms and strategies that reduced the impact of stressors.	Societal and organizational stressors: Public incivility, media representation, demanding work environment, lack of appreciation, and inadequate training and protocols were identified as key stressors. Burnout mitigation: Organizational well-being services were beneficial to some, but not all, due to accessibility and appropriateness issues. Peer discussions and positive public feedback were found to bolster well-being. Recommendations: Call handlers suggested improvements like adequate breaks, involvement in training and protocol design, and informal discussions to address everyday stressors.	Two experienced qualitative researchers independently coded every transcript and met regularly to discuss differences, enhancing the reliability of findings.	n = 17 of the 18 participants worked in one of two 999-EMS operations center sites, which may introduce bias into the results. The study planned to recruit a sample of n = 20; the authors do not state whether data saturation was achieved at n = 18 participants or whether these were the only participants recruited.	No further relevant studies.
Record-Jackson (2022) [61], USA	Resilience and Coping among First Responders in the Central Valley	ProQuest	Quantitative	One large ambulance service in the USA	EMT, paramedics, and ambulance behavioral health team	n = 106	Evaluate how critical incidents are experienced and represented by the individual and within the profession	Cross-sectional survey	Descriptive and inferential statistics and qualitative narrative of open-ended questions (no analysis platform specified)	The results of the study can be classified into three main areas: (1) the need to receive emotional support from colleagues, (2) individual coping strategies implemented, and (3) participants’ perceptions of peer support as a resource that could facilitate processing of personal experiences and feelings concerning subjectively complex events on an emotional level.	The authors identified a number of factors that may influence coping mechanisms to inform future research.	A small sample from one EMS organization may not represent the broader population of first responders. Moving from a paper to an online survey format during the study led to technical issues and attrition, particularly in ranking coping strategies, hampering the reliability. How open-ended survey questions were analyzed and how themes were constructed are not described.	No further relevant studies.
Swab (2019) [62], USA	Stress Management of EMS Providers	ProQuest	Mixed methods	One large ambulance service in the USA	EMS providers (EMT, AEMT, EMT-P, PHPE, and prehospital physician)	n = 153	Identify positive and negative influences that could predicate better stress management leading to healthier lifestyles	Self-constructed online questionnaire (using QuestionPro® software) based on a review of the literature and influence of Barber et al.’s (2015) [44] study	Descriptive statistics using SPSS and narrative coding of open-ended survey questions	The study found that stress in EMS providers is exacerbated by stigma and long shifts but highlighted the benefits of stress management education, resources, supportive administration, and critical incident stress management (CISM).	The methods are underpinned by relevant theory (Pearlin’s Stress Model, 1989).	A small sample and low response rate from one EMS organization may not represent the broader population of EMS providers.	No further relevant studies.
Tessier et al. (2021) [32], Canada	Adherence to Psychological First Aid (PFA) after Exposure to a Traumatic Event at Work among EMS Workers: A Qualitative Study	Database	Qualitative	One large ambulance service in Canada	Paramedics and EMDs trained as peer helpers	n = 11 (n = 9 paramedics and n = 2 EMDs)	Qualitatively identify factors that influence the adherence of peer helpers and recipients to psychological first aid intervention in an EMS organization by exploring peer helper perspectives.	Semi-structured telephone interviews	Inductive thematic analysis, using QDA Miner 5.0 software package	(1) Individual perceptions and attitudes of peer helpers and recipients about PFA intervention, (2) perceived impacts on peer helpers and recipients, (3) organizational support to PFA intervention, and (4) congruence with the occupational culture. Study findings suggest that it is conceivable to act on various factors to improve adherence to PFA intervention among peer helpers and recipients within EMS organizations. This could lead to enhanced understanding of the challenges involved in sustaining a peer-led PFA program for first responders.	One interviewer and information power were employed as strategies to ensure the reliability and transferability of results.	The report does not provide specific details about the context in which PFA was implemented or the specific traumatic events encountered.	No further relevant studies. Mental health stigma and barriers to mental health care for first responders: A systematic review and meta-analysis, https://doi.org/10.1016/j.jpsychires.2017.08.001, (accessed on 20 February 2024) was read and excluded as the sample was <50% prehospital EMS.
Tunks-Leach et al. (2021) [63], Australia	The Role and Value of Chaplains in the Ambulance Service, https://doi.org/10.1007/s10943-021-01446-9 (accessed on 6 January 2024).	MedNar	Qualitative	One large Australian EMS organization (New South Wales)	Paramedics	n = 17	Paramedic perspectives on the role and value of chaplains in the ambulance service	Semi-structured interviews (first phase of sequential mixed-methods approach)	Framework analysis	Paramedics valued ambulance chaplains for their emotional and spiritual support, which helped them cope with stress and trauma. The importance of organizational factors in the chaplains’ effectiveness was emphasized.	Generating and refining the interview questions following a scoping review and pilot interviews enhanced the internal validity of the findings.	There is potential for response bias as paramedics who have had positive experiences with chaplains may be more likely to participate in the study. Whether analysis was undertaken by one or more independent researchers is not reported, hampering the replicability and reliability of the results. The semi-structured interview guide is not included, making it hard to determine whether data collection methods could be replicated.	No further relevant studies.
Witczak-Bloszyk et al. (2022) [50], Poland	Work-Related Suicide Exposure, Occupational Burnout, and Coping in Emergency Medical Services Personnel in Poland	Database	Quantitative	Poland	EMS employees	n = 411 (paramedics (71.8%), nurses (15.8%), medical doctors (9.0%), and other medical professionals, such as radiologists (3.4%))	Psychological aftermath of suicide exposure in EMS in Poland	Online questionnaire: 30-item custom-designed questionnaire section (demographics, workplace suicide exposure, availability of workplace psychological support, and informal social support); including the Link Burnout Questionnaire (LBQ) and Coping Inventory for stressful situations (CISS)	Descriptive, inferential and partial squares modelling	EMS personnel are frequently exposed to suicide as part of their professional activities. The LBQ score indicated symptoms of burnout, in particular relational deterioration, and the CISS showed low levels of emotion-oriented coping. Physicians reported higher levels of psycho-physical exhaustion than paramedics and nurses. Access to psychological support in the workplace was related to lower levels of burnout.	The methodology that utilized established scales worked well to indicate work-related suicide exposure, burnout, and coping mechanisms among emergency medical services personnel in Poland. The study demonstrated that EMS employees were willing to complete a questionnaire about this subject.	The term “work-related suicide exposure” has been conceptualized in terms of frequencies and/or dichotomized “yes/no” types of exposure, which might have oversimplified this complex variable, which could include completed suicide, suicide ideation, or self-harm depending upon participant perceptions.	No further relevant studies.
Williams et al. (2023) [52], UK	Practical psychosocial care for providers of pre-hospital care: a summary of the report ‘valuing staff, valuing patients’	Database	WP1: Review of clinical, scientific, managerial, and policy sources WP2: systematic review to describe current knowledge of the psychiatric and psychosocial consequences of working in pre-hospital care, and to identify any factors that could be causative or contribute to these impacts WP3: members of the team conducted extensive fieldwork by visiting practitioners and services and attending conferences and training events. As part of its fieldwork, the team took evidence from the Pre-hospital Emergency Medicine Trainees Association (PHEMTA) surveys of PHEMTA members	Seven academic databases: MEDLINE, PubMed, PsycINFO, CINAHL, ProQuest, the Cochrane Library, and HMIC. The following relevant journals were hand-searched for references: *Trauma*, *Journal of Traumatic Stress*, *Journal of Loss and Trauma: International Perspectives on Stress & Coping*, *Traumatology: An International Journal*, *Emergency Medicine Journal*.	Clinical or non-clinical employees working in pre-hospital medical care, such as ambulance crews, pre-hospital physicians, and dispatchers.	Systematic review included n = 156 papers and n = 13 dissertations and theses. Sample size/characteristics not provided. EMS employee “interviews/conversations” = no sample size provided.	Summarize current knowledge of the psychiatric and psychosocial consequences of working in pre-hospital care and identify any factors that could be causative or contribute to these impacts.	Systematic review and employee interviews	Narrative reports on themes identified in qualitative studies, and factors causing or contributing to psychosocial problems. Series of consensus meetings where findings are agreed upon and refined; qual or quant analysis methods are not reported, expert opinion only.	Secondary stressors and adverse outcomes can be modified by the adequacy and effectiveness of employers’ responses to events and expectations of employees’ performance, career aspirations, and concerns of staff about their training, the conditions in which they work and live, and their work–life balance. Fifteen key approaches are suggested for EMS organizations to care for the well-being, psychosocial, and mental health needs of their employees.	The study takes a multidisciplinary approach, combining scholarship, research, and fieldwork. This holistic perspective enhances the credibility of its findings and recommendations.	Analysis methods not described: In neither the PROSPERO record nor this article was how themes were derived (other than consensus meetings held by the research team) explained. The report does not explore how contextual factors (such as geographical location, cultural differences, or organizational structures) may influence the effectiveness of the proposed recommendations.	A further paper detailing the methods was identified in the reference list and was reviewed in tandem with this summary paper to inform the quality appraisal: https://fphc.rcsed.ac.uk/media/3140/valuing-staff-valuing-patients.pdf (accessed on 7 April 2025)
