# Supplementary material for: Why Do Emergency Medical Service Employees (Not) Seek Organizational Help for Mental Health Support?: A Systematic Review
Source: Int J Environ Res Public Health. 2025 Apr 17;22(4):629. doi: 10.3390/ijerph22040629 (PMC12027444; doi:10.3390/ijerph22040629)
Supplement: Supplementary file 1 [file ijerph-22-00629-s001.zip › Supplementary Material List.docx]

Summary list of Supplementary Materials

| **In Text Order** | **Reference** | **Content** | **Page No.** |
| --- | --- | --- | --- |
|  | Supplementary Material S1 | Search strategy | 3 |
|  | Supplementary Material S2 | Table S1: Webpages searched | 3 |
|  | Supplementary Material S3 [In text] | Table S2: Inclusion and exclusion criteria | 4 |
|  | Supplementary Material S4 | Methods: Reflexivity and Theoretical Underpinning | 4 |
|  | Supplementary Material S5 [In text] | Figure S1: PRISMA Flow diagram | 6 |
|  | Supplementary Material S6 | Table S3: TIDiER checklist results | 7 |
|  | Supplementary Material S7 | Table S4: Quality assessment of included studies | 7 |
|  | Supplementary Material S8 | Figure S2: Word cloud visual representation | 7 |
|  | Supplementary Material S9 | Table S5: Overview of themes and associated codes | 8 |
|  | Supplementary Material S10 | Table S6: Illustrative quotes supporting identified themes | 8 |
|  | Supplementary Material S11 | Table S7: Assessment of codes following sensitivity analysis | 13 |
