# Supplementary material for: Why Do Emergency Medical Service Employees (Not) Seek Organizational Help for Mental Health Support?: A Systematic Review
Source: Int J Environ Res Public Health. 2025 Apr 17;22(4):629. doi: 10.3390/ijerph22040629 (PMC12027444; doi:10.3390/ijerph22040629)
Supplement: Supplementary file 1 [file ijerph-22-00629-s001.zip › Supplementary Material S1—Search strategy.docx]

**Supplementary Material S1:** Search strategy

This search strategy was developed in partnership with expert librarians from the Bodleian Library (University of Oxford, UK) and professional National Health Service (NHS) library services (Discovery Library, Plymouth, UK).

**(Title/Abstract) (Medline):**

1. “pre hospital"
2. pre-hospital
3. prehospital
4. paramedic*
5. ambulance*
6. aeromedical
7. “Aviation medicine”
8. HEMS
9. helicopter ADJ5 emergenc*
10. EMT
11. "emergency medical" ADJ technician*
12. ECA
13. "emergency care" ADJ assistant*
14. "Emergency call" ADJ (handler* OR operator*)
15. "Emergency dispatch*"
16. #1 OR #2 OR #3 OR #4 OR #5 OR #6 OR #7 OR #8 OR #9 OR #10 OR #11 OR #12 OR #13 OR #14 OR #15
17. anxiety
18. depress*
19. ptsd
20. "post-traumatic stress"
21. "post traumatic stress"
22. burnout
23. "burn out"
24. "self-harm"
25. self harm
26. "self injur*"
27. self-injur*
28. "self mutilat*"
29. self-mutilat*
30. distress
31. "mental health"
32. "mental illness*"
33. well-being
34. wellbeing
35. stress*
36. suicid*
37. "critical incident stress"
38. #17 OR #18 OR #19 OR #20 OR #21 OR #22 OR #23 OR #34 OR #25 OR #26 OR #27 OR #28 OR #29 OR #30 OR #31 OR #32 OR #33 OR #34OR #35 OR #36 OR #37
39. "occupational health"
40. "occupational mental health"
41. "occupational support"
42. "psychological support"
43. "psychological help"
44. help-seeking
45. help ADJ3 seeking
46. signposting
47. "employee assistance"
48. "employee support"
49. resilience
50. organi?ation* ADJ3 (support OR assistance)
51. work* ADJ3 (support OR assistance)
52. manager* ADJ3 (support OR assistance)
53. "crisis intervention"
54. downtime
55. surveil*
56. monitor*
57. #38 OR #39 OR #40 OR #41 OR #42 OR #43 OR #44 OR # 45 OR #46 OR #47 OR #48 OR #49 OR # 50 OR #51 OR #52 OR # 53 OR # 54 OR #55 OR #56
58. #16 AND #38 AND #57
