# Supplementary material for: Why Do Emergency Medical Service Employees (Not) Seek Organizational Help for Mental Health Support?: A Systematic Review
Source: Int J Environ Res Public Health. 2025 Apr 17;22(4):629. doi: 10.3390/ijerph22040629 (PMC12027444; doi:10.3390/ijerph22040629)
Supplement: Supplementary file 1 [file ijerph-22-00629-s001.zip › Supplementary Material S2—Table S1 Webpages searched.docx]

**Supplementary Material S2, Table S1:** List of webpages searched for grey literature

| **Organization** | **Website address** |
| --- | --- |
| The Ambulance Staff Charity (UK) | <https://www.theasc.org.uk/>  <https://www.rightsteps.co.uk/customers/tasc> |
| The Royal Foundation (UK) | https://royalfoundation.com/mental-health/ |
| The mental health charity Mind  (Mind Bluelight / Mind together) (UK) | https://bluelighttogether.org.uk/ambulance/ |
| Paramedic association of Canada | https://paramedic.ca/resources/documents |
| Association of Ambulance Chief Executives (AACE) (UK & Ireland) (including the Global Ambulance Leadership Alliance) (UK) | https://aace.org.uk/ |
| International roundtable on community paramedicine (Canada) | https://ircp.info/ |
| National EMS Management Association (USA) | https://www.nemsma.org/ |
| Paramedic Chiefs of Canada (Canada) | https://www.paramedicchiefs.ca/ |
| Council of Ambulance Authorities (AUS/NZL/PNG) | https://www.caa.net.au/ |
| British Columbia Emergency Health Services (Canada) | http://www.bcehs.ca |
| EMS Denmark | https://www.regionh.dk/ |
| National Ambulance Resilience Unit (NARU) (UK) | https://naru.org.uk/ |
