# Supplementary material for: Why Do Emergency Medical Service Employees (Not) Seek Organizational Help for Mental Health Support?: A Systematic Review
Source: Int J Environ Res Public Health. 2025 Apr 17;22(4):629. doi: 10.3390/ijerph22040629 (PMC12027444; doi:10.3390/ijerph22040629)
Supplement: Supplementary file 1 [file ijerph-22-00629-s001.zip › Supplementary Material S3—Table S2 Inclusion and exclusion criteria.docx]

Supplementary Materials S3, Table S2: Inclusion and exclusion criteria

| **Inclusion (Population, Phenomena of Interest, Context, and Types (PICoT))** | **Exclusion** |
| --- | --- |
| Population: Adults (18+) employed by government/state commissioned EMS ambulance services *. Eligible employees contracted to full- or part-time roles or holding a bank/non-substantive contract requiring regular working hours.  Phenomena of Interest: Organizational interventions offered to support EMS employee mental health and any perceived barriers to and/ or enablers of utilizing such support.  Context**:** Government/state commissioned EMS ambulance organizations.  All Types of study designs, including qualitative, quantitative, and gray literature published from 1st December 2004 onward. | 1. Patient-/general population-focused: not EMS employees. 2. Sample not state funded EMS.** (private and charity ambulance services excluded from review). 3. Sample <50% EMS (or mixed emergency responders/private ambulance workers where % numbers are not defined). 4. Physical (including fatigue/sleep) not mental health. 5. Barriers to and/or enablers of support not examined/discussed. 6. Disaster/conflict/major incident response. 7. Physical not psychological working environment. 8. Social (friends/family) not organizational support. 9. Population is student/volunteer/bystander/apprentice/family and friends of EMS workers.** 10. Full text not available (or not available in English). 11. RCT/review/study protocol or interim report. 12. Scale validation study. |

* Employees could include paramedics, emergency medical technicians, emergency care assistants, EMS ambulance nurses and doctors, emergency medical number call center and dispatch employees, operational managers, support, and central function employees such as human resources and patient safety teams, as well as senior leadership who work for out-of-hospital emergency medical ambulance services. ** These groups are excluded as available supportive interventions may differ from those offered to employed staff.
