# Supplementary material for: Why Do Emergency Medical Service Employees (Not) Seek Organizational Help for Mental Health Support?: A Systematic Review
Source: Int J Environ Res Public Health. 2025 Apr 17;22(4):629. doi: 10.3390/ijerph22040629 (PMC12027444; doi:10.3390/ijerph22040629)
Supplement: Supplementary file 1 [file ijerph-22-00629-s001.zip › Supplementary Material S4—Methods.docx]

**Supplementary Material S4,** **Methods**: Reflexivity and theoretical underpinning.

**Reflexivity**

Reflexivity was woven throughout the methodological process and included exploration of whether the authors’ personal and professional experiences of EMS, research, and/or mental health support influenced our understanding of barriers and facilitators faced by EMS employees. Throughout the study, reflective journaling, documenting of thoughts, decision-making, peer-to-peer discussion, and acknowledgment of bias encouraged the researchers to critically examine assumptions, values, and interpretation of the data. This process helped to identify potential blind spots, whilst encouraging consideration of alternative explanations for any observed patterns. The authors worked in or were familiar with the fields of mental health and emergency services through professional background and previous research. We acknowledge that these insights may lead to thematic preferences and prioritization of themes that may align with professional interests or individual experiences. Reflexivity encouraged awareness and discussion about such inclinations and prompted consideration of alternative interpretations and exploration of themes beyond our initial instincts. The continual process of evaluating whether codes were semantic or latent, further aided reflexivity. The maintenance of a latent code decision-log augmented transparency, which underpins systematic and reflexive research methods. An example of such reflexivity during the coding process, extracted from the lead author’s reflexive journal, follows:

“As I analyze the articles, it is interesting that a number of studies speak about how EMS employees want to/prefer to speak to organizational peer support networks about the psychological residue of their work, however data also stated that ‘*ESWs often avoided talking to their colleagues about their emotions following traumatic calls*.’ There is clearly a disconnection between what staff want and what they feel they can actually do. I noticed that after reading this, my experience as a paramedic influenced my interpretation of the data – my empathy towards their struggles and anxiety about connecting with colleagues led me to focus more on their emotional responses, rather than some of the more structural and strategy-based aspects of barriers and facilitators to utilizing organizational support. Acknowledging and bracketing this bias helped me to adjust my approach and ensured a more focused and balanced perspective in my analysis.”

**Theoretical Underpinning**

Our procedure was underpinned by a realist approach which acknowledged that the reality of the findings existed independently of our views as researchers but accepted that we played a role in constructing the knowledge. Two authors constructed themes using a reflexive thematic analysis (RTA) viewed through an experiential lens [29]. This approach was selected to describe ambulance employees’ experiences and perspectives about seeking organizational help for mental health support, while respecting the external reality in which they inhabit and providing an explanation for these factors, which is as a key aspect of realism. Reflexive Thematic analysis allowed us to explore semantic (explicit) and latent (implicit) content. By minimizing researcher interpretation (whilst still acknowledging the researcher’s active role in knowledge production), we aligned with the inductive nature of RTA, by avoiding the imposition of pre-existing assumptions. By predominantly capturing semantic meaning whilst recognizing the potential for latent codes, we maintained openness to participants' experiences and the underlying reality whilst constructing the data.
