# Supplementary material for: Why Do Emergency Medical Service Employees (Not) Seek Organizational Help for Mental Health Support?: A Systematic Review
Source: Int J Environ Res Public Health. 2025 Apr 17;22(4):629. doi: 10.3390/ijerph22040629 (PMC12027444; doi:10.3390/ijerph22040629)
Supplement: Supplementary file 1 [file ijerph-22-00629-s001.zip › Supplementary Material S6—Table S3 TIDiER Checklist.docx]

**Supplementary Material S6, Table S3**: TIDieR checklist summary of interventions described in included articles

| **Study ID** | **Intervention name** | **Why** | **WHAT (Materials)** | **Procedures** | **Who Provided** | **How** | **Where** | **When &** **How much** | **Tailoring** | **Modifications** | **How well-planned** | **How well - actual** |
| --- | --- | --- | --- | --- | --- | --- | --- | --- | --- | --- | --- | --- |
| Adams et al, (2014)  Australia | Priority One at Queensland Ambulance Service. | Promotes good mental health and wellbeing, informs and empowers ambulance staff to access resources for them and their families. | A multi-layered staff support service, aiming to fill the gaps in supportive services for staff and their families. | Provides psychosocial and resilience education across the organization. There is a point of contact for all stakeholders, including managers and those leading other support services, to provide advice and supervision. | MDT included internal counsellors, a Director, and a Chaplain. The team also included psychologists and registered counsellors, some who had frontline ambulance experience. Specific training included critical incident management, resilience, and suicide. | Self-referral for counselling online. | In the workplace and virtually. | Not Specified | Not Specified | Not Specified | Not Specified | Not Specified |
| Al-Wathinani et al, (2023)  Saudi Arabia | No named intervention | N/A | N/A | N/A | N/A | N/A | N/A | N/A | N/A | N/A | N/A | N/A |
| Alzahrani et al, (2017)  Saudi Arabia | PSU | Provides psychological support, prevention, and treatment. | Brochures summarizing PSU sent via email to raise awareness. | As part of the research project, the referral process changed to individual referrals to PSU directly, rather than being referred through multiple hierarchical levels. | Not Specified | Electronic Consultation available. | Main office. | Not Specified | Not Specified | Sending brochures with information on the PSU and its services via emails to all employees. Introduced electronic consultation services for staff. Adapted the referral process, see ‘Procedures’. | Not Specified | Not Specified |
| Auth et al, (2022)  United Kingdom | (1) Single Session CISD (2) TRiM (3) Schwartz rounds (4) Blue Light Champions | (1) Preventing PTSD (2) A peer support system. (3) Improve staff psychological well-being and increase empathy and compassion. (4) Blue Light Champions challenge stigma and increase understanding of mental health in the workplace. They are also said to be more aware, responsive, and equipped to deal with their own and their colleagues’ mental health needs. | (3) Sharing difficult thoughts, feelings, and experiences with others in a connected environment. (4) Raises awareness of mental health problems and challenges the stigma. Lead Blue Light Champions oversee and coordinate the Champions, and an Internal Worker group comprises of individuals across the service. | (4) No preset sequence of steps. Facilitators were encouraged to be creative in their role, which depended upon how much time they had to give to the role. If they have more time, they could become a point of contact for staff and volunteers. | (4) Any current employee or volunteer could become a Blue Light Champion. It was a voluntary role and there can be any number of Champions in a service. It was also suggested that Champions could discuss with Line Managers whether they could have dedicated time for the role within their shift. | (4) Variable depending on level of engagement and time Blue Light Champion has to give to the role. 1:1 talks to organizational wide anti-stigma education. | (4) In the organization. | (4) variable depending on activities chosen by Blue Light Champion and wider internal working group. | Not Specified | Not Specified | Not Specified | Not Specified |
| Barber et al, (2015)  USA | (1) EAP. (2) CISM. | (1) EAP was an employee benefit program for a range of difficulties. (2) CISM is a debriefing procedure to help manage and normalize incidents witnessed in the workplace. | CISM includes pre- and post-incident support, and crisis intervention. | -CISD  -Defusing -Grief and Loss Sessions -Crisis Management Briefings  -Critical Incident Adjustment Support  -Pre-Crisis Education  - CISM Aftercare from trauma counsellors via telephone. | Trained CISM personnel from an external service. | 1:1, in structured groups, varying in length depending on program chosen as previously described. | In person and via telephone. | Not Specified | Not Specified | Not Specified | Not Specified | Not Specified |
| Canadian Standards Association (2018)  Canada | (1) EAP (2) Peer support programs (3) Return-to-work and stay-at-work programs (4) Risk mitigation process (5) Referral to external mental health professionals (6) Psychological wellness checks (7) Supervisor contact (8) Early identification system (9) Psychoeducation on mental health (10) Resilience skills-building programs (11) Proactive outreach to those exhibiting concerning changes in behavior (12) Post-trauma support (unspecified) (13) Self-care promotion and support. (14) Stigma reduction program (15) Suicide prevention/awareness program (16) Identification and strengthening of protective factors (17) Inclusion of psychological health and safety support in critical and emergency plans (both organizational and individual). (18) Creation of a dedicated intranet site for staff with information on relevant psychological health and safety policies, programs, and resources. (19) Development of an integrated psychological health program for staff (20) Critical Incident Stress Management program (21) Psychoeducation for family members of EMS workers | (2) Peer support is effective in preventing OSIs and improving quality of life. (6) Psychological wellness checks can help identify risks and provide recommendations for paramedics’ mental health. (17) To ensure that psychological support is considered. | Not Specified | (3) Regular follow-up meetings should be scheduled to review the workers' progress, ensure their needs are met and make the necessary modifications. (4) The organization develops and maintains the risk mitigation process. (6) A confidential discussion between workers and a mental health professional about their work and potential psychological risks. (9) Provide information about factors in the workplace that contribute to psychological health and safety. (15) Evidence-informed programs on suicide awareness, prevention, intervention, and postvention programs must be maintained by the service. (16) Identify hazards and risk factors and strengthen protective factors. (19) Training in resilience, team building and psychological safety skills, plus access to online self-care modules on various topics including depression and substance misuse. (20) Shifting from team debriefing to an improved incident reporting process, including a range of individual or group options. | (2) Trained peer supporters. (3) All management and worker representatives need training. (4) Management (5) Adequately trained external mental health professionals (e.g., psychologists, counsellors). (6) A regulated mental health professional should conduct wellness checks, organized by the paramedic service. (7) Supervisor(s) (8) Management (9) Train-the-trainer opportunities can enhance mental health support for paramedics. (14) The paramedic service organization. (16) Paramedic service organizations. | (2) Peer support programs should be implemented as part of regular routine. (22) Face to face, group format | Not Specified | (3) Following return to work after an absence. (6) As part of induction/orientation to establish a baseline, and follow-up checks to assess coping skills thereafter. (9) Induction through to retirement, regularly scheduled. (12) Post-trauma, both early and ongoing. | (3) Individualized, flexible, graduated, and modifiable. | Not Specified | Use of the PHSMS (Psychological Health and Safety Management System) involves auditing and feedback relevant to policy adherence, data collection, and intervention effectiveness. | Not Specified |
| Carvello et al (2019)  Italy | (1) Resilience in Stressful Events - Peer support Program (2) Defusing/emotional first aid (3) AREU peer support program (4) Psychologist Support | Not Specified | Not Specified | 1)Peer Supporters call colleagues within 30 minutes, focusing on emotions rather than technical details. Calls are confidential, except in cases of imminent harm. 3) Active listening | (1) Healthcare professionals (2) the “defuser” is trained in empathic listening (3) Peers who are trained and assisted by health emergency psychologists. | (1) Face to face or via telephone. | Not Specified | (1) Within half an hour of receiving the request. | Not Specified | Not Specified | Not Specified | Not Specified |
| Clompus and Albarran (2016)  United Kingdom | No named intervention | N/A | N/A | N/A | N/A | N/A | N/A | N/A | N/A | N/A | N/A | N/A |
| Coyte et al (2024)  Australia | No named intervention | N/A | N/A | N/A | N/A | N/A | N/A | N/A | N/A | N/A | N/A | N/A |
| Fischer and Macphee (2017)  Canada | (1) Psychologists and Psychiatrists, particularly those who have been trained to understand the paramedic environment. (2) EAPs (3) Peer support networks | Not Specified | Not Specified | Not Specified | Not Specified | Not Specified | Paramedics have to travel to another location to access counselling services. | Upper limit of 8 counselling sessions available to some paramedics due to insurance limitations | Not Specified | Not Specified | Not Specified | Not Specified |
| Gallagher and Mcgilloway, (2007)  Ireland | Peer Support Program under CISM. | CISM is a psychosocial support program. | Use of leaflets and posters around the work base to advertise the Peer Support Program | - Assessment for defusing.  - Provides short-term peer counselling.  - Liaises with other peer supporters. | Training lasts 3 days, and can be completed virtually, online at your own pace, or in person. | 1:1 and in groups. | At the work base. | Not Specified | Not Specified | Not Specified | Not Specified | Not Specified |
| Gouweloos-Trines et al, (2017)  Eight Western Industrialized Countries | Formal Peer Support. | To listen, identify those at risk, and provide low intensity psychological intervention. | Peer support is integrated into day-to-day working practice. Peer supporters do not have 'clients' that they see on an ongoing basis as these conversations tend to involve signposting and offering alternative referral pathways. However, there may be times where individuals require support for longer periods, such as for specific populations like veterans. | Employees are offered their Peer Supporter as an initial point of contact following exposure. Employees can also select their Peer Supporter. | Trained colleagues, with extensive experience, should undergo assessment and training. Training should include basic skills needed for the role such as listening skills, psychological first aid and information about referral processes, but should not extend to interventive training such as cognitive restructuring. Mental health professionals should also provide supervision and training. | Not Specified | Not Specified | Peer support to be offered as the initial point of contact after the event. Can be spontaneous and informal, at any time of the working day. | Not Specified | Not Specified | Not Specified | Not Specified |
| Hadas (2019)  USA | (1) Peer support groups, (2) CISM, (3) After-action debriefings, (4) Counsellors, (5) EAP | Not Specified | Not Specified | Not Specified | Not Specified | Not Specified | Not Specified | Not Specified | Not Specified | Not Specified | Not Specified | Not Specified |
| Halpern et al, (2008)  Canada | (1) Supervisor emotional support, (2) Timeout | (1) to listen and provide material support.  (2) A time to decompress/relax before starting paperwork | (1) a discussion with supervisor, not described as structured. Limited information available. (2) A time to speak about the incident, at their own pace, in a comfortable environment. | (1) Provided in the immediate aftermath of the incident.  (2) Typically requested by the individual, but sometimes offered by supervisors. ½-1 hours. | (1) Supervisor.  (2) Supervisors may suggest or facilitate the time out by taking EMT worker for a coffee or break from working environment. | Not Specified | (1) in working base/telephone.  (2) no specific environment | (1) in the first 24 hours after the incident. (2) immediately after incident, lasting 1-2 hours. | Not Specified | Not Specified | Not Specified | Not Specified |
| Halpern et al, (2009)  Canada | (1) CISD (2) Peer support (3) Supervisor support (4) A brief period post-critical incident in which to access support. (5) Educational initiatives such as Morbidity and Mortality Rounds (6) Education on signs and symptoms that they may require further support following a critical incident for EMS workers and their family members. | Not Specified | Not Specified | Not Specified | Not Specified | Not Specified | Not Specified | Not Specified | Not Specified | Not Specified | Not Specified | Not Specified |
| Hugelius et al, (2014)  Sweden | "Crisis Intervention Session" - Single session group meetings, regarded as similar to debriefing | To discuss their actions at the scene of the traumatic incident, and how they felt about the situation. | Followed a predefined structure | Single structured session, post traumatic event. | Led by the Ambulance Manager or Senior Colleague | Group, in person | At their workplace | Varied from as soon as possible after the incident, later the same day, or the morning after. | Structured, no tailoring suggestions. | Not Specified | Once a year meeting with Crisis Support Managers to monitor good and bad experiences. | Not Specified |
| Jackson et al, (2017)  United Kingdom | TRiM | A peer-to-peer screening tool to identify stress symptoms and refer to professional counselling services when needed. It aims to improve accessibility and reduce stigma by providing 1-month follow up assessments post traumatic incident via peer support. | Questions and scoring that make up the structured risk assessment are provided. Logbooks used to capture all activity related to TRiM. | Individuals are invited to take part in a risk assessment following a major incident including all relevant staff and families. Individuals can also request a TRiM assessment, or their line manager can submit them for an assessment with their consent.  1 month of "active monitoring" and then a second risk assessment is completed, which is compared with the 72-hour risk assessment to see how the individual is coping. Those not improving are referred for specialist support. | Peer Assessors are Operational Personnel, trained in delivering psychological risk assessments and basic level of trauma psychology. Managers are also trained as practitioners or peer assessors first and are then responsible for running the TRiM program and support practitioners. Training course during via Strong Mind Resilience. One day refresher training is also available, but Managers can complete this with their teams informally. | Provided 1:1 or in groups. Session is guided by the practitioner/peer assessor through structured interview questions, whilst also being described as informal. | Not Specified | A structured risk assessment post traumatic incident, and another assessment roughly one month after the incident. | Tailored depending on outcomes at 1month assessment. E.g., onward referrals or suggestions to managers about changing working timetable. Cultural differences and language difficulties are considered. | Not Specified | Through training assessments to assess competence. | CPD Logbooks exist to monitor up to date training and refresher courses and adhere to TRiM Code of Conduct and Standards |
| Kellner et al, (2019)  Australia | Support from frontline managers | To reduce the severity of post-traumatic symptoms | Not Specified | Conversational support. Peer support officers check in on staff exposed to particularly difficult or traumatic events. | Frontline managers | Face-to-face and telephone. Individual and group. | Not Specified | Following traumatic events. | Not Specified | Not Specified | Not Specified | Not Specified |
| Kling and Ripley, (2020)  United Kingdom | (1) Post-critical incident debriefings (2) Educational training sessions (in e.g., burnout prevention/stress management) (3) Mental health support (4) Peer mentorships (5) Nutritional classes (6) Gym and exercise activity (7) Supervisor mentorships (8) EMS chaplains (9) Mindfulness training | Investigation to explore the needs of EMS individuals to aid prediction of the needs of EMS personnel. | Survey | (1) No information provided. (2) 'to teach coping skills to adjust to the difficulties of the EMS occupation' (3-8) No information provided. | Not Specified | Not Specified | Not Specified | Not Specified | Not Specified | Not Specified | Not Specified | Not Specified |
| Lawn et al, (2020)  Australia | Multiple named interventions: (1) debriefing or mandatory crisis meetings; (2) cognitive behavioral therapy; (3) peer support programs; (4) psychological first aid | Not Specified | Not Specified | Not Specified | Not Specified | Not Specified | Not Specified | Not Specified | Not Specified | Not Specified | Not Specified | Not Specified |
| Lilly et al, (2019)  USA and Canada | Destress 9-1-1, an online mindfulness-based intervention. | To reduce stress through the repeated practice of mindfulness. The aim was to reduce stress levels and improve mindfulness 'scores' post intervention and at 3 months follow up. The intervention was informed by MBSR. | Baseline survey. Online intervention comprising of 7 modules, each contain short videos, guided meditation, mindfulness activities, and daily check ins.  A stress reduction toolkit for call centers is made available post intervention to all managers. | Email invites sent from call center administrators. Destress 9-1-1 involved a 7-week online intervention. Two were emails sent each week; one introducing the week's theme/content, and one as a reminder. Post treatment survey completed and debriefed online. | Online intervention with no practitioner assistance. The individuals that created the intervention were clinicians trained in mindfulness-based approaches. | Online | Online | 7 modules, one to be completed every week for 7 weeks. Each module typically lasted 20-30 minutes. Progress was tracked. | Not Specified | MBSR was modified for this study-shortened, made available online, and tailored to the needs of 911 call out centers. | Adherence to treatment measured by module completion rates, and preset reminders sent out each week to each individual completing Destress 911. | . Not Specified |
| Loudoun et al, (2020)  Australia | (1) Peer support officers (2) EAP | Not Specified | Not Specified | Formal EAPs can vary in their composition across organizations but typically include debriefing/defusing sessions, peer support officer (PSO) programs, pre‐incident and post‐incident education and training, telephone services and professional counsellors. | (1) Peers in the EMS (2) Peer supporters, professional counsellors. | (1) Face-to-face | In the vehicle on the way back to base or in communal areas at their station. | Often in the immediate aftermath of traumatic experiences. | Not Specified | Not Specified | Not Specified | Not Specified |
| Mackinnon et al, (2022)  Australia | No named intervention | N/A | N/A | N/A | N/A | N/A | N/A | N/A | N/A | N/A | N/A | N/A |
| National EMS Management Association (USA) (2016)  USA | (1) EAP (2) Critical Incident Stress Counselling (3) Chaplaincy programs (4) Conflict resolution programs (5) Peer support programs (6) Substance abuse program (7) Crisis hotline access (8) Mental health awareness training (9) Social worker or therapist on staff (10) Screening, brief intervention and referral to treatment (11) Resiliency training (12) Referral to external mental health services (not part of the EAP). (13) Access to fitness centers (14) Tobacco cessation programs (15) Stress management classes (16) Dietary and nutrition counselling (17) Access to a health clinic on premises (18) Group exercise class (19) Access to a fitness center either on site or locally | Not Specified | Survey | (1) EAP elements can include family counselling, legal help, smoking cessation, and stress management programs. | (1) EAPs are usually staffed by licensed professionals, including clinical social workers, mental health counselors and substance abuse professionals. (3) Chaplains. (9) Social worker or therapist on staff. | Not Specified | Not Specified | (1) Counselling provided as part of EAPs is usually short-term (6 free sessions is the norm). Sometimes just three sessions are provided. | Not Specified | Not Specified | Not Specified | Not Specified |
| Ntatamala and Adams, (2022)  South  Africa | (1) Training on how to manage work-related stress (2) Support from management (3) Counselling (4) At-risk workers should be followed-up | Factors associated with increased risk for PTSD in ambulance personnel and the barriers faced in accessing support for work-related stress. | Survey | Not Specified | Not Specified | (3) Counselling could be delivered over the telephone or, preferably, face-to-face | (3) Counselling would ideally be on work premises if delivered face-to-face | Not Specified | Not Specified | Not Specified | Not Specified | Not Specified |
| Paramedic Chiefs of Canada (2014)  Canada | (1)E(F)APs. (2) CISM. (3) Chaplaincy/spiritual counselling. (4) Peer Support. (5) Psychoeducation. | (all) To reduce the severity of post-traumatic symptoms | Not Specified | Employees needing help are identified by field supervisors. Diffusion and debriefing are offered. Staff are trained in psychological stress awareness. There is specific mental health training for managers. There is also on-call support for crises/critical incidents. Some trusts had both baseline and ongoing measurement of staff psychological health. Plus, staff at risk of greater psychological stress/harm based on certain types of calls or call volume/rate are flagged. Some trusts had a referral network for passing cases on to external services. | (2) CISM coordinators. Peer nomination, interview, and psychological screening to select members of the peer resource team, followed by training (3) Chaplaincy counsellors.  . | Individual and group. | Various locations in the ambulance working context, including in the field, at hospital and ambulance stations. | (2) Staff attend CISM program following a critical incident, the same happens with defusing and debriefing. Some psychoeducation/training in mental health and job-related stress is part of standard orientations. | Not Specified | Not Specified | Not Specified | Not Specified |
| Phung et al, (2022)  United Kingdom | (1) TRiM. (2) Counselling. (3) CBT | Not Specified | Not Specified | Not Specified | Not Specified | Not Specified | Not Specified | Not Specified | Not Specified | Not Specified | Not Specified | Not Specified |
| Powell et al, (2023)  United Kingdom | Occupational Health referrals to counselling. PIC. A button call handlers could press to gain support during their shift. Peer Support. Helplines. | Not Specified | Not Specified | Not Specified | Not Specified | Not Specified | Not Specified | Not Specified | Not Specified | Not Specified | Not Specified | Not Specified |
| Record-Jackson (2022)  USA | (1) CISD (2) Peer support (3) Signposting to external therapy (4) Helplines (5) Employee Assistance Programs (6) Chaplains | (1) CISD was intended to introduce a standard way of managing the emotional/mental trauma of critical incidents due to a recognition of concerning levels of harm and trauma in first responders. | Not Specified | Not Specified | Not Specified | Not Specified | Not Specified | Not Specified | Not Specified | Not Specified | Not Specified | Not Specified |
| Swab  (2019)  USA | (1) Critical Incident Stress Debriefing/Management (2) Psychological First Aid (3) Education on stress management (4) EAP (5) Companioning (6) Structured Peer Support Programs (7) Behavioral Health First Aid (8) Signposting to externally delivered therapy/counselling (9) Support from administration (10) Reduced membership rates to gyms or other exercise facilities (11) Access to music and religious/spiritual resources, e.g. chaplaincy programs | (1) CISM was intended to assist first responders with the emotional burdens of their job. | Not Specified | (1) EMS workers discuss traumatic events, receive psychoeducation, and coping tips in groups. Counselors offer additional support. (2) Crisis intervention process identifying those needing assistance visually or behaviorally. Includes safety, information gathering, practical help, and social support. (4) Employers provide free guidance and counseling for personal or work-related issues. (5) Connects emotionally distressed individuals with active listeners.  ( 9) Encourages staff to seek help and fosters an open atmosphere for discussing problems with management. | (1) 'Trained facilitator' for CISD/M. (5) Active listeners. (6) Volunteers with no specific training. (8) External, trained therapists/counsellors (9) Administrative staff | Face-to-face or in groups. | (2) It is designed to be delivered anywhere. | (1) Following traumatic events (24-72 hours later). (2) Following or 'immediately following a traumatizing incident. (3) Some mandatory training, with optional training available. Certification by the National Registry of Emergency Medical Technicians does not mandate training in stress management. | Not Specified | Not Specified | Not Specified | Not Specified |
| Tessier et al (2021)  Canada | PFA, an early intervention approach. | Reduce initial distress and promote coping strategies after a traumatic event. Coping Strategies to be used in short and long term.  *Information gathered from email exchange*: *"PFA is based on a number of experts in the fields of trauma and disasters* *whom have identified the following five empirically-supported intervention principles to guide intervention practices following disaster and mass violence at the early to mid-term stages."* | The Brymer training manual includes 8 Core Actions with a goal within each. The Training Manual goes into detail about how to approach each core action and suggestions on how to structure/what to say. Appendix also includes worksheets that could be used. | PFA is provided in the early hours after the event, once the EMS have returned to the station. | Peer Helpers, who are EMS workers. Minimal training in mental health required. 8-10 Peer Helps are trained in groups (14-hours) using Brymers PFA Manual, facilitated by the organizations Psychologist. All had at least 10 years of professional experience as EMS. | Can be delivered 1:1 or in groups. Mostly face-to-face intervention but can be done on the telephone or online. | When the EMS workers returned to the organizations site. Offered use of a room or environment. Although also noted that it can be performed anywhere. | In the immediate aftermath of disasters. Schedule described as flexible, and amount of time spent on each of the 8 core actions should be specific to the individuals' specific needs. Follow ups vary depending on the severity of difficulties. Each meeting is 20 minutes – 1 hour in length. | Some Peer Helpers described that they could be flexible with order of the 8 core actions. The Brymer Manual, gives tailoring suggestions for working with individuals with disabilities and is described as culturally informed. | Not Specified | PFA providers should feel confident in the interventions and principle of confidentiality, and organizational procedures should be put in place, including regular supervision and training follow-ups. | Ongoing training on PFA core actions and additional training to ensure quality of interventions remains high. However, Peer Helpers recognize more could be done to track what is working well/what is not to be reflective and improve. |
| Tunks-Leach et al, (2021)  Australia | Chaplain support for ambulance staff | Chaplains play a crucial role in supporting paramedics’ emotional, spiritual, and psychological well-being. | Not Specified | Chaplains offer holistic conversational support to ambulance staff, addressing emotional well-being. They build relationships, normalize help-seeking, and assist in post-incident support. | Chaplains (multi- or inter-faith spiritual care practitioners experienced in supporting staff). | In-person face-to-face in the ambulance working environment | Conversations with chaplains occurred in a number of settings, such as in the ambulance, being taken for coffee or at community events like BBQs. | Number of times the intervention was delivered was flexible depending upon chaplain availability and staff need | The intervention is personalized, providing conversational support tailored to individual needs rather than following a standardized approach. | Not Specified | Not Specified | Not Specified |
| Williams et al, (2023)  United Kingdom | (1) PFA (2) Peer Support; (3) TRiM, (4) Occupational Health Services, (OH). | (1) recognizes basic needs as a priority. (2) provides opportunity for a supportive relationship between people with commonalities. (4) improving health and wellbeing, improving productivity. | Not Specified | (1) Basic objectives described as establishing a compassionate connection, providing an immediate and continued sense of safety, provide a comforting and calm environment, recognizing someone’s immediate needs and offering practical support and information such as services and signposting. (4) Prevention, early intervention, rehabilitation, teaching and training. | Not Specified | (1) one to one. | Not Specified | (1)During and after the incident. | Not Specified | Not Specified | Not Specified | Not Specified |
| Witczak-Bloszyk et al, (2022)  Poland | (1) CISD (2) Workplace psychological support | Not Specified | Not Specified | Not Specified | (2) A trained and regulated psychologist in the workplace, providing support as part of their normal role. | Not Specified | Not Specified | Not Specified | Not Specified | Not Specified | Not Specified | Not Specified |
| **Key:** | | | | | | | | | | | | |
| AREU – Regional Emergency Urgency Association  CISD - Critical Incident Stress Debriefing  CISM - Critical Incident Stress Management  EAP - Employee Assistance Programs  EMS – Emergency Medical Service | | | | E(F)APs - Employee (and Family) Assistance Programs  MBSR - Mindfulness-Based Stress Reduction  MDT - Multidisciplinary Team.  OSI – Occupational Stress Injury  PFA – Psychological First Aid | | | | PHSMS - Psychological Health and Safety Management System  PSU - Psychological Support Unit  PSW - Peer Support Worker  PTSD - Post Traumatic Stress Disorder  TRiM – Trauma Risk Management | | | | |
