# Supplementary material for: Why Do Emergency Medical Service Employees (Not) Seek Organizational Help for Mental Health Support?: A Systematic Review
Source: Int J Environ Res Public Health. 2025 Apr 17;22(4):629. doi: 10.3390/ijerph22040629 (PMC12027444; doi:10.3390/ijerph22040629)
Supplement: Supplementary file 1 [file ijerph-22-00629-s001.zip › Supplementary Material S7—Table S4 Quality assessment.docx]

**Supplementary Material S7, Table S4:** Quality assessment of included studies

|  | **Included Article** | | **Checklist**  **applied** | **Checklist Questions** | | | | | | | | | | | | | | **Quality**  **Overall** | **Sensitivity analysis** |
| --- | --- | --- | --- | --- | --- | --- | --- | --- | --- | --- | --- | --- | --- | --- | --- | --- | --- | --- | --- |
|  | **Study ID** | **Title** |  | **1** | **2** | **3** | **4** | **5** | **6** | **7** | **8** | **9** | **10** | **11** | **12** | **13** | **14** |  |  |
|  | Adams et al (2015)  Australia | An Interpretative Phenomenological Analysis of Stress and Well-Being in Emergency Medical Dispatchers. | Qual | Yes | Yes | Yes | Yes | Yes | Yes | Yes | Partial | Yes | No |  |  |  |  | Low Risk | n/a |
|  | Al-Wathinani et al (2023)  Saudi Arabia | Enhancing Psychological Resilience:  Examining the Impact of Managerial Support on Mental Health Outcomes for Saudi Ambulance Personnel. | Quant | Yes | Yes | Yes | Yes | n/a | n/a | n/a | Yes | Yes | Yes | Yes | n/a | Yes | Partial | Unclear | n/a |
|  | Alzahrani et al (2017)  Saudi Arabia | Improving referral to psychological support unit at Saudi Red Crescent  Authority in Riyadh Region. | Grey | Yes | Partial | Yes | Yes | Yes | Yes |  |  |  |  |  |  |  |  | Unclear | n/a |
|  | Auth et al(2022)  UK | Mental health and help seeking among trauma-exposed emergency service staff: a qualitative evidence synthesis. | SR | Yes | Yes | Yes | Yes | Yes | Yes | Yes | Yes | No | Yes | Yes |  |  |  | Low Risk | n/a |
|  | Canadian Standards Association (2018) | Psychological health and safety in the paramedic service organization. | Grey | Yes | No | Yes | Partial | Yes | Yes |  |  |  |  |  |  |  |  | High Risk | Removed |
|  | Carvello et al (2019)  Italy | Peer-support: a coping strategy for nurses working at the Emergency  Ambulance Service. | Qual | Yes | Partial | Yes | Yes | Yes | Yes | Partial | No | Partial | No |  |  |  |  | Unclear | n/a |
|  | Clompus et al (2016)  UK | Exploring the nature of resilience in paramedic practice: A psycho-social study. | Qual | Yes | Yes | Yes | Yes | Yes | Partial | Yes | Yes | Yes | No |  |  |  |  | Low Risk | n/a |
|  | Coyte et al (2024)  Australia | Resilience, posttraumatic growth, and psychological well-being of paramedicine clinicians: An integrative review. | SR | Yes | Yes | Yes | Yes | Yes | Yes | Yes | Yes | No | Yes | Yes |  |  |  | Low Risk | n/a |
|  | Fischer et al (2017) Canada | Canadian paramedic health and  wellness project: Workforce profile and health and wellness trends. | Qual | Yes | Partial | Yes | Yes | Partial | Partial | Yes | Yes | Yes | No |  |  |  |  | Low Risk | n/a |
|  |  |  | Quant | Yes | Yes | Yes | Yes | n/a | n/a | n/a | Yes | Yes | Yes | No | n/a | Yes | Yes | Low Risk | n/a |
|  | Gallagher et al (2007)  Ireland | Living in critical times: The impact of critical incidents on frontline  ambulance personnel:  a qualitative perspective. | Qual | Yes | Yes | Yes | Yes | Partial | Yes | Partial | No | Partial | No |  |  |  |  | Unclear | n/a |
|  | Gouweloos-Trines et al (2017)  Multiple | Perceived support at work after critical incidents and its relation to  psychological distress: a survey among prehospital providers. | Quant | Yes | Yes | Partial | Yes | n/a | n/a | n/a | Yes | Partial | Yes | Yes | n/a | Yes | Yes | Low Risk | n/a |
|  | Hadas (2019) USA | Affects of Mental Health Limitations, Leadership Interactions, and Generational Diversity on the Morale of  Paramedics in Public and Private  Emergency Medical Services. | Qual | Yes | Yes | Yes | Partial | Yes | Yes | Partial | Yes | Yes | No |  |  |  |  | Low Risk | n/a |
|  |  |  | Quant | Yes | Partial | Partial | Partial | n/a | n/a | n/a | Partial | n/a | Partial | Partial | n/a | Partial | Partial | Unclear |  |
|  | Halpern et al (2008)  Canada | Interventions for critical incident stress in emergency medical services: A  qualitative study. | Qual | Yes | Yes | Yes | Yes | Partial | Yes | Yes | Partial | Yes | No |  |  |  |  | Unclear | n/a |
|  | Halpern et al (2009)  Canada | What makes an incident critical for ambulance workers? Emotional outcomes and implications for intervention. | Qual | Yes | Yes | Yes | Yes | Yes | Yes | Yes | Yes | Yes | No |  |  |  |  | Low Risk | n/a |
|  | Hugelius et al (2014)  Sweden | Swedish Ambulance Managers’  Descriptions of Crisis Support for  Ambulance Staff After Potentially  Traumatic Events. | Qual | Yes | Yes | Yes | Yes | Yes | Yes | Yes | No | Yes | No |  |  |  |  | Low Risk | n/a |
|  | Kellner et al (2019) Australia | BARRIERS TO FRONTLINE  MANAGER SUPPORT FOR HIGH-TRAUMA WORKERS | Qual | Yes | Yes | Yes | Yes | Yes | Partial | Yes | No | Yes | No |  |  |  |  | Low Risk | n/a |
|  | Kling (2020)  UK | Needs Assessment for Mental Health Support Towards Emergency Medical Service (EMS) Personnel. | Quant | Yes | Yes | Yes | Yes | n/a | n/a | n/a | Partial | Partial | Yes | n/a | n/a | Yes | Yes | Low Risk | n/a |
|  | Jackson et al (2017)  UK | Optimizing Workplace Support to Manage Stress and Improve Health. | Grey | Yes | Partial | Yes | Partial | Yes | Partial |  |  |  |  |  |  |  |  | High Risk | Removed |
|  | Lawn et al (2020) Australia | The effects of emergency medical  service work on the psychological, physical, and social well-being of ambulance personnel: a systematic review of qualitative research. | SR | Yes | Yes | Yes | Yes | Yes | No | Partial | Yes | No | Yes | Yes |  |  |  | Unclear | n/a |
|  | Lilly et al (2019) USA &  Canada | Destress 9-1-1—an online mindfulness-based intervention in reducing stress among emergency medical dispatchers: a randomized controlled trial. | RCT | Yes | No | Yes | No | No | Yes | Partial | Yes | Yes | Yes | Yes | Yes | Yes |  | Unclear | n/a |
|  | Loudoun et al (2020)  Australia | The role of peer-to-peer voice in severe work environments: organizational facilitators and barriers. | Qual | Yes | Yes | Yes | Yes | Yes | Yes | Yes | No | Yes | No |  |  |  |  | Low Risk | n/a |
|  | Mackinnon et al (2020)  Australia | Risk of psychological distress, pervasiveness of stigma and utilization of support services: Exploring paramedic perceptions. | Quant | Yes | Yes | Yes | Yes | n/a | n/a | n/a | Yes | Partial | Yes | Yes | n/a | Yes | Partial | Low Risk | n/a |
|  | National EMS Management Association (USA) (2016) | NATIONAL SURVEY ON EMS MENTAL HEALTH SERVICES. | Quant | Yes | Partial | Partial | No | n/a | n/a | n/a | Partial | Partial | No | No | n/a | Partial | Partial | High Risk | Removed |
|  | Barber et al (2015)  USA | Survey Reveals Alarming Rates of EMS Provider Stress and Thoughts of Suicide. | Quant | Yes | Partial | Partial | No | n/a | n/a | n/a | Partial | Partial | No | No | n/a | Partial | Partial | High Risk | Removed |
|  | Ntatamala et al (2022)  South Africa | The Correlates of Post-Traumatic Stress Disorder in Ambulance Personnel and Barriers Faced in Accessing Care for Work-Related Stress. | Quant | Yes | Yes | Yes | Yes | n/a | n/a | n/a | Partial | Partial | Yes | Yes | n/a | Partial | Partial | Low Risk | n/a |
|  | Paramedic Chiefs of Canada (2014) | Operational Stress Injury in Paramedic Services: A Briefing to the Paramedic Chiefs of Canada. | Grey | Yes | No | Yes | Partial | Yes | Yes |  |  |  |  |  |  |  |  | High Risk | Removed |
|  | Phung et al (2022)  UK | The experiences and perceptions of well-being provision among English ambulance services staff: a multi-method qualitative study. | Qual | Yes | Yes | Yes | Yes | Yes | Yes | Yes | No | Yes | No |  |  |  |  | Low Risk | n/a |
|  | Powell et al (2023)  UK | A qualitative analysis of stressors affecting 999 ambulance call handlers' mental health and well-being. | Qual | Yes | Yes | Yes | Yes | Yes | Yes | Partial | Partial | Yes | Partial |  |  |  |  | Low Risk | n/a |
|  | Record-Jackson (2022) USA | Resilience and Coping among First Responders in the Central Valley. | Quant | Partial | Yes | Partial | Partial | n/a | n/a | n/a | Partial | Partial | Partial | No | n/a | Partial | Partial | High Risk | Removed |
|  | Swab (2019) USA | Stress Management of EMS Providers. | Qual | Yes | Yes | Yes | Yes | Partial | Yes | Partial | No | Partial | Partial |  |  |  |  | Unclear | n/a |
|  |  |  | Quant | Yes | Yes | Yes | Yes | n/a | n/a | n/a | Partial | Partial | Partial | No | n/a | Yes | Yes | Low Risk | n/a |
|  | Tessier et al (2021)  Canada | Adherence to Psychological First Aid after Exposure to a Traumatic Event at Work among EMS Workers: A Qualitative Study. | Qual | Yes | Yes | Yes | Yes | Yes | Partial | Yes | Yes | Yes | No |  |  |  |  | Low Risk | n/a |
|  | Tunks-Leach et al (2021)  Australia | The Role and Value of Chaplains in the Ambulance Service: Paramedic Perspectives. | Qual | Yes | Yes | Yes | Yes | Yes | Yes | Partial | No | Yes | Yes |  |  |  |  | Low Risk | n/a |
|  | Witczak-Bloszyk et al (2022)  Poland | Work-Related Suicide Exposure, Occupational Burnout, and Coping in Emergency Medical Services Personnel in Poland. | Grey | Yes | Yes | Yes | Partial | Yes | Yes |  |  |  |  |  |  |  |  | Unclear | n/a |
|  | Williams et al (2023)  UK | Practical psychosocial care for providers of pre-hospital care: a summary of the report ‘valuing staff, valuing  patients. | SR | Yes | Yes | Yes | Yes | Yes | Yes | Yes | Yes | No | Yes | Yes |  |  |  | Low Risk | n/a |
