# Supplementary material for: Why Do Emergency Medical Service Employees (Not) Seek Organizational Help for Mental Health Support?: A Systematic Review
Source: Int J Environ Res Public Health. 2025 Apr 17;22(4):629. doi: 10.3390/ijerph22040629 (PMC12027444; doi:10.3390/ijerph22040629)

**Supplementary Material** **S8, Figure S2***:* Visual representation (Word Cloud) of underlying data generated using NVivo 12 software


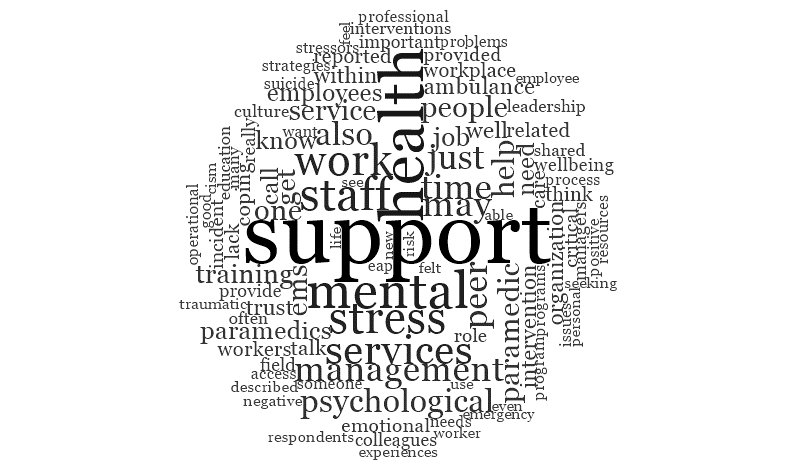

Supplement: Supplementary file 1 [file ijerph-22-00629-s001.zip › Supplementary Material S8—Figure S2 Word cloud.docx]
