# Supplementary material for: Why Do Emergency Medical Service Employees (Not) Seek Organizational Help for Mental Health Support?: A Systematic Review
Source: Int J Environ Res Public Health. 2025 Apr 17;22(4):629. doi: 10.3390/ijerph22040629 (PMC12027444; doi:10.3390/ijerph22040629)
Supplement: Supplementary file 1 [file ijerph-22-00629-s001.zip › Supplementary Material S9—Table S5 Overview of themes and codes.docx]

**Supplementary Material S9, Table** **S5**: Overview of themes and associated codes

|  | **Predominant theme:** | **Subtheme** | **Subtheme** | **Subtheme** |
| --- | --- | --- | --- | --- |
| **Themes** | **Organizational culture that shapes how employees interact, make decisions, and approach their mental health at work.** | **Employee (in)ability to talk about**  **mental health and ask for help.** | **Provision and utilization of person-**  **centered support tailored to the EMS context.** | **Nurture staff and the organization through education and training about recognition, disclosure, and support**  **utilization for mental health.** |
| **Theme short name** | **Culture** | **Employee** | **Support** | **Training** |
| **Barriers** | **Article/s reporting barriers** | | | |
|  | (31) 1-5, 7-10, 12-24 and 26-34 | (32) 1-4, 6-14 and 16-34 | (33) 1-27 and 29-34 | (9) 3,7,8,9,10,12-14,23,32 |
| **Employee factors** | Perception of organizational obligation rather than genuine care.  Employees can feel expendable and unvalued.  Distressing experiences are unrecognized or not acknowledged by the organization. | Fear of disclosure due to stigma, fear of appearing weak to colleagues, and potential for negative career consequences.  Maladaptive Coping mechanisms such as avoidant coping.  Delayed psychological impact.  Poor employee mental health.  Caring for self, conflicts with sense of duty.  Lack of awareness about available support | Lack of support that is easily accessible, timely, and useful.  Unable to afford support.  Lack of adequate and choice of support based on individual need.  Lack of confidence in the effectiveness of available interventions.  Lack of information about available services.  Previous negative experiences inform future participation. | Inadequate employee training.  Lack of training and skill building for managers. |
| **Support**  **delivery**  **factors** | Insufficient time and funding are allocated for employee support.  Manager support can be inconsistent, task-orientated (rather than people-orientated) and underpinned by a perceived lack of empathy. | Taking time out to support self or colleagues can be perceived as conflicting with assigned job responsibilities. | Delivering/facilitating support can be incompatible with assigned job responsibilities, especially for peer supporters.  Additional emotional and workload burden for peer facilitators.  Lack of competent support facilitators and mental health professionals who understand the EMS context.  Competence, skills, and emotional unsuitability of managers to provide support. | Lack of time at work for training and education about employee mental health. |
| **Organisational Factors** | Stigma about mental health results in fear of being judged and appearing weak, and of negative career consequences.  Organisational culture that stigmatizes and discourages open discourse about employee mental health and respects stoicism.  Machismo and a brotherhood of masculine, uniformed cultural norms, where stress and mental health is not talked about.  Lack of understanding and commitment about the importance of providing time and balancing employee needs against operational needs.  Lack of organizational strategy and culture to look after the ‘whole person’ at work.  Poor communication about mental health at work. | Poor working relationships between colleagues.  Feeling uncomfortable to speak openly in front of peers.  Stigmatizing attitudes held by leaders.  Fear of disciplinary procedures for taking time out for mental health discussion and support.  Perceived lack of trust, discretion, and confidentiality  In a rule-bound work environment, the lack of active organizational encouragement to discuss and utilize workplace support prevents some individuals from seeking help and sharing their needs. | Lack of suicide prevention despite known risk.  Inconsistent language and terminology.  Formal debriefing about clinical incidents was seen as unhelpful and as harmful by some.  Lack of a robust evidence base to underpin what supportive interventions should be offered.  Requiring employees to pay rather than providing free of charge support. | Reactionary organizational culture and commitment to mental health  Employees feel that they are not involved in decision-making.  Lack of a robust evidence base to underpin training. |
| **Enablers** | **Article/s reporting the theme.** |  |  |  |
|  | **Culture** | **Employee** | **Support** | **Training** |
|  | (31) 1-4, 7-30 and 32-24 | (32) 1-5, 7-14. 16-34 | (29) 2-4, 6, 8-14, 16-17 and 19-34 | (20) 4-7, 14-15, 17-18, 20-21, 25-26, 30-31 |
| **Employee factors** | Employees know and believe in organizational goals, mission, and values relating to employee mental health and well-being .  Cultural norms include looking after self and colleagues. | Feeling safe to disclose and feeling heard.  Sense of belonging and shared sense of purpose.  Feeling valued by the organization.  Self-awareness and adaptive coping-mechanisms  Speaking with peers due to shared humor and camaraderie.  Speaking with mental health experts who understand the EMS context.  Confidentiality. | Improved mental health by enabling employees to recognize symptoms early, manage stress effectively, and maintain psychological resilience.  Belief that the support offered will help.  Proactive, preventative support –Being asked rather than plucking up the courage to speak. | Education for employees to recognize symptoms of acute and cumulative stress and distress in themselves and their colleagues.  Emotional awareness and preparedness education.  Training to build confidence and knowledge for disclosure and uptake of support services. |
| **Support**  **delivery**  **factors** | Genuine and committed leadership who lead by example for employee mental health.  valuing and prioritizing preventive support for all employees (opposed to reactive services for those already distressed). | Flexibility and time at work to respond to employee needs.  Renumeration for those facilitating support activities (in particular peer supporters).  Peer to peer debriefing following clinical incidents. | Proactive support – reaching out to employees rather than standing by.  Willingness to look and listen.  Prevention for all rather than reactive services for those already distressed.  Effective interventions that consider the use of cognitive reappraisal.  Robust evidence-base, fostered through high-quality, context specific research.  Support delivered by trained, confident and non-judgmental persons who understand the context.  Regular support and Clinical Supervision for those delivering support. | High quality intervention delivery training  Brief and efficient training.  Train and educate employees, managers and friends and family. |
| **Organisational Factors** | The organization sets the tone by:  providing time at work to proactively look and listen for opportunity to support employee mental health.  Developing systems designed to look after the whole person.  Integrated systems, workforce policy, and planning that is sensitive to, prioritizes, and encourages employee mental health discourse and support utilization.  Joint accountability for employee well-being – it takes a village.  Stakeholder engagement to inform decision-making. | Being actively encouraged by management and culture to disclose and uptake support.  Critical incident experiences recognized and acknowledged by the organization.  Symptoms of poor mental health recognized by colleagues and meet with genuine concern and support.  Knowing what support is available and how it can help. | A range of adequate, useful, and easy to deliver support options.  Person-centered support that is tailored to job role risk and is inclusive and representative of the workforce population.  Strengthening of confidence in the effectiveness of available interventions.  Timely and accessible support available when the employee is ready to talk.  Support tailored to and delivered by those who understand the EMS context. | Organisational level training and readiness for employee mental health and well-being .  Increased research and education, to inform organizational understanding about the risk posed by different workforce roles.  Evaluate and measure the effectiveness of support provided. |
| **Both barrier and enabler** | **Article/s reporting the theme.** | | | |
|  | **Culture** | **Employee** | **Support** | **Training** |
|  | (13) 3, 8, 10, 12, 16, 19-21, 23-24, 27, 29, 30 | (26) 1-4, 8, 10, 12-14, 16-19, 21, 23-34 | (11) 8, 10, 12-14, 23-26, 29, 32 | 0 |
| **Employee factors** |  | Pre-ambulance life experiences (such as traumatic childhood events).  Work-related incidents impact individuals differently.  Influence of length of service:   - newly qualified employees, who may not yet built-up trust in peers and the organization, are more likely to seek support outside of work from friends and family - Experienced employees are more likely to utilize organizational support as worry about burdening friends and family by sharing work-related experiences but are less likely to find it useful. | Adequacy of organizational response to employee psychological needs mediates outcomes. |  |
| **Support**  **delivery**  **factors** |  |  | Competence and confidence of the person/s delivering the support can enhance or hamper the quality and meaningfulness of supportive interventions.  Provision of mandatory support can reduce stigma but may be met with resistance and reduce intervention effectiveness. |  |
| **Organisational Factors** | Patient care – can hamper time at work for support delivery but can also be influenced by employee mental health.  Communication can promote or create barriers and tension to a healthy culture. |  |  |  |

Highlighted sections – Factors left unsupported following sensitivity analysis
