# Supplementary material for: Why Do Emergency Medical Service Employees (Not) Seek Organizational Help for Mental Health Support?: A Systematic Review
Source: Int J Environ Res Public Health. 2025 Apr 17;22(4):629. doi: 10.3390/ijerph22040629 (PMC12027444; doi:10.3390/ijerph22040629)
Supplement: Supplementary file 1 [file ijerph-22-00629-s001.zip › Supplementary Material S10—Table S6 Illustrative quotes.docx]

**Supplementary material S10, Table S6:** Illustrative quotes supporting identified themes

| **Quote** | **Study** |
| --- | --- |
| **Overarching Theme: Organizational Culture** | |
| "This is a silent epidemic. I have seen people depart the profession and have seen profound damage done to others… You can’t hand somebody an EAP card and expect a result" | (Swab, 2019) |
| “Over a fifth feared that seeking help for work related stress would negatively affect their career” | (Ntatamala and Adams, 2022) |
| “Initiating mental health services through the EAP is an invitation for mandatory competency evaluation, grounds for dismissal” | (Goodwin et al*.*, 2016) |
| “..if they think you’re suffering from stress, then they think you shouldn’t be doing the job. If you can’t handle it, get out!” | (Gallagher and McGilloway, 2007) |
| “This was compounded if reports were not taken seriously or were under-reported, as culturally, potential aggression and violence was seen as a ‘normal part of the job” | (Lawn et al*.*, 2020) |
| “paramedics also felt unable to disclose feelings, both from a desire to shield others from distress, but also because of shame associated with masculine-dominated workplace culture, where feelings were not discussed” | (Goodwin et al*.*, 2016) |
| "The agency I work for sees mental health as a weakness. If you ask for help you become verbally abused by co-workers, supervisors and station managers. I needed help and was told, ‘that’s why women don’t belong in EMS. They’re overly emotional…" | (Swab, 2019) |
| “A common sentiment heard from EMS practitioners is that mental health issues aren’t something people want to talk about, for fear of being seen as weak or soft, or at worse, unfit to do their jobs” | (Gallagher and McGilloway, 2007) |
| “Accessing mental health resources was stigmatized as being weak and unfit for duty” | (Fischer and Macphee, 2017) |
| “They [dispatch] have no idea what it’s like to be on the road and they don’t care. They just care about getting our response times down.” | (Fischer and Macphee, 2017) |
| “I think people just need to be treated like professionals. Paramedics think we just take calls, they have no idea’’ | (Adams, Shakespeare-Finch and Armstrong, 2015) |
| “We are unwanted, unsupported, unheard, overwhelmed and overworked. It's not about patient care or taking care of people anymore. It's all about money and trucks (ambulances) in service” | (Swab, 2019) |
| **Subtheme 1: Employee (in)ability to talk about mental health and ask for help** | |
| “Workplace culture contributed to reluctance to disclose negative emotions” | (Coyte et al*.*, 2023) |
| “..these services had their origins in paramilitary culture, with a strong hierarchical chain of command, which in-turn prizes stoicism in the face of adversity..” | (Lawn et al., 2020) |
| “When time-out opportunities were unavailable, ESWs describe rushing into the next call without having psychologically processed the previous call” | (Auth et al*.*, 2022) |
| “Being able to access mental health or general health and wellbeing services while on duty may make it more feasible for EMS practitioners to actually make use of them. This is not a common practice in EMS” | (Goodwin et al*.*, 2016) |
| "Most of the people in my organization do not feel comfortable using any service provided by the organization for fear that the information will come back and be used against them in the future" | (Goodwin et al*.*, 2016) |
| “If we were to see genuine concern from our managers, from our immediate supervisors and they punctually show up and ask how you’re doing, how you’re feeling, that goes a long way!” | (Halpern et al*.*, 2009b) |
| “We all went out on a works do . . .. There were so many of us that were in this group out who had had awful, or what could be deemed as awful, upbringings” | (Clompus and Albarran, 2016) |
| “Many of the participants related that they felt their administration was apathetic and indifferent in handling the potential impact that the suicide could have had on staff and operations” | (Swab, 2019) |
| “discussions among the management showed that even the so-called "old guard" (those who may believe that paramedics should be psychologically toughened) support putting operational stress injury programs in place” | (Ad-hoc Committee on Operational Stress Injury, 2014) |
| “In new recruits especially, who have not developed a strong connection with peers or who are fearful of appearing weak or incompetent, family and friends are the preferred companions” | (Halpern et al*.*, 2009a) |
| “One interviewee suggested that an incident which caused him significant distress early in his career was more impactful because he had not yet established a base of support within the workplace” | (Halpern et al*.*, 2009b) |
| “for more experienced EMTs, family and friends are often protected from the ‘burden’ of hearing about critical incidents” | (Halpern et al*.*, 2009a) |
| “I've been doing this a long time and I am on the road but I also wear three pips on the shoulder. I can be empathetic but I'm not and in my role - you've got to talk to your level…” | (Kellner et al*.*, 2019) |
| **Subtheme 2: Provision and utilization of person-centered support tailored to the EMS context** | |
| "The few debriefings I have attended over 22 years, they all bring back up what we are desperately trying to let go of or bury" | (Goodwin et al*.*, 2016) |
| “There is evidence that ‘debriefing’ of this nature has the potential to cause harm and that it should be avoided” | (Williams et al*.*, 2022) |
| “Support provided by management is sporadic and, on occasion, still incorporates the use of the single debrief session. This method has little or no evidence supporting its use. Furthermore, the practice can actually further embed negative feelings and lead to resentment.” | (Jackson and Romano, 2017) |
| “Paramedics reported that organisational support and peer networks were invaluable to help process emotions” | (Coyte et al*.*, 2023) |
| “I started full-time in '94 and every day a shift was fifty per cent of day in vehicle and fifty per cent in the station. So, you could put that into training & debrief time.. It's really only the older staff that remember it.. the new grads know nothing else but busy.” | (Loudoun et al*.*, 2020) |
| “For the union, there is a recognition that the peer-to-peer voice is necessary, but being eroded through the escalating expectations on their members, which means ‘it's just from job to job to job.” | (Loudoun et al*.*, 2020) |
| “The only thing I regret is that no one asked me: “Do you want to talk about it with someone? Do you need to talk to someone about your emergency intervention?” ... "I kept my experience inside, I elaborated the event by myself.” | (Carvello et al*.*, 2019) |
| “Intervention strategies require systems to identify those who may be at risk of stress injury, while prevention blankets large groups of mostly healthy people with tactics designed to reduce their exposure to stress and/or the risk of injury from stress.” | (Ad-hoc Committee on Operational Stress Injury, 2014) |
| “A lot of people don’t like ... selecting that button because it creates too much attention and I’ll put my hand up – I’ve used it once in the 12 months I’ve been here but there’s been numerous times when, instead of clicking that button, I’ve clicked on a comfort break ... because I just don’t want the attention ... sometimes we’re that busy with call stacking, I’d feel a bit guilty ... I’m a bit of an old fashioned kind of guy ... I don’t really talk about mental health” | (Powell et al*.*, 2022) |
| “Responses that are not quick may be perceived by emergency responders as taking too long” | (Ad-hoc Committee on Operational Stress Injury, 2014) |
| “XS16 went on to share that each one of the individuals that attempted suicide had reached out prior to the event” | (Hadas, 2019) |
| “I’ve actually had a psychologist who said, ‘yes, I know what you mean’. No, you don’t. Or, ‘I know how you feel’. You haven’t got a clue how I feel..” | (Tunks Leach et al*.*, 2022) |
| “EMS Practitioners want counsellors who understand the job of EMS. General counsellors, therapists or psychologists who are uninformed about EMS are seen as offering little useful help.” | (Goodwin et al*.*, 2016) |
| “To cope with daily calls from people experiencing trauma and crisis, participants described how they used cognitive reappraisals to construct new narratives that hold meaning to them and assist them to make sense of their emotions, their role, and their relationship with the caller.” | (Adams, Shakespeare-Finch and Armstrong, 2015) |
| "You have to attend for other colleagues; that has never been a problem, you participate, and that’s about respect" | (Hugelius et al*.*, 2014) |
| “the support effectiveness was greater when the provider attended on a voluntary basis versus instances where a field provider was mandated to attend.” | (Newland et al*.*, 2015) |
| “..the feeling of an abundance or lack of support directly affects the quality of care delivered to the patient” | (Jackson and Romano, 2017) |
| **Subtheme 3: Education and training** | |
| "Mental health should be a fundamental part of the EMS training curriculum so that EMTs, paramedics, first responders all know what it is, how to identify it, how to deal with stress and where to get help" | (Goodwin et al*.*, 2016) |
| “Open-ended survey questions revealed a dominant theme of deficiency in the management of stress in EMS professionals: a lack of stress management education” | (Swab, 2019) |
| “I understand that at times decisions have to be made, but it seems that a majority of the time the decisions are opposite of what is being asked for by the employees” | (Hadas, 2019) |
| “He went on to share that in the fire department all the employees knew what the departments goals, mission and values were. Not only did they know, they believed in it because they were each an important voice in creating them” | (Hadas, 2019) |
| “There was strong evidence the major obstacle to quality support across cases was lack of training for Frontline Manager’s … “the intent may never be for team leaders to be the first point of call for that trauma counselling, they just are” | (Kellner et al*.*, 2019) |
| “Emergency Service Worker’s expressed a desire to be informed about the types of emotions which could be triggered by work-related traumatic incidents, which may reduce shame associated with help seeking” | (Auth et al., 2022) |
| “almost seventy percent of participants had no training, whilst almost thirty percent had some training and less than three percent had extensive training in how to manage work related stress” | (Ntatamala and Adams, 2022) |
| “Very few EMS agencies have set benchmarks or measure outcomes related to the effectiveness of mental health services” | (Goodwin et al., 2016) |
| “Employees may also not want to answer truthfully because they fear their responses will be shared. And if too few answer the survey, the results may not reflect what’s really going on” | (Goodwin et al*.*, 2016) |
| “Once a year all people trained to lead crisis support meetings gather together with the head of ambulance services to discuss good and bad experiences, and that can be a slap in the face for stations with less adequately functioning crisis support” | (Hugelius et al*.*, 2014) |
