# Supplementary material for: Why Do Emergency Medical Service Employees (Not) Seek Organizational Help for Mental Health Support?: A Systematic Review
Source: Int J Environ Res Public Health. 2025 Apr 17;22(4):629. doi: 10.3390/ijerph22040629 (PMC12027444; doi:10.3390/ijerph22040629)
Supplement: Supplementary file 1 [file ijerph-22-00629-s001.zip › Supplementary Material S11—Table S7 Sensitivity analysis.docx]

**Supplementary Material S11, Table S7:** Assessment of codes following sensitivity analysis

| **Theme** | **Code name** | **Before sensitivity analysis (n=)** | | **After sensitivity analysis (n=)** | | **Supported by**  **(article/s)** | **Assessment** | **Consequences for constructed themes** |
| --- | --- | --- | --- | --- | --- | --- | --- | --- |
|  |  | Article | Refs | Article | Refs |  |  |  |
| **Barriers** | Whole theme | 34 | 1035 | 28 | 832 | All | The overall volume of associated references was reduced by one fifth. However, low-quality articles and their associated references were distributed evenly across the nodes and themes. | Sensitivity analysis demonstrated that the removal of low-quality studies had little impact on barriers and although this affected the weight of support, the constructed themes still reflected the underlying data. |
| **Both** | **Staff / Employee factors** / Low engagement | 1 | 1 | 0 | 0 | **16** | A small factor included in the child node of ‘*employee perception’*, the removal of this information has little impact on the ‘higher level ‘*employee factors’* or ‘*perceptions*’ node. | None |
|  | **Friends and Family** / Access to organizational support | 1 | 2 | 0 | 0 | **29** | Providing friends and family with education is no longer supported (the strength of support was weak before) | These are all small factors which support why friends and family should be involved in organizational planning when considering how to support staff, however, the strength of this information is weak and is unsupported by higher quality studies – further research would be recommended to examine the usefulness of this approach for enabling staff to recognize symptoms and access organizational support when needed. |
|  | Communication | 1 | 1 | 0 | 0 | **16** | Comms about MH extended to family members is no longer supported (the strength of support was weak before) |  |
|  | Early warning systems | 1 | 1 | 0 | 0 | **16** | The organization using friends and family as an early warning system is no longer supported (the strength of support was weak before) |  |
|  | Education | 1 | 2 | 0 | 0 | **16** | The organization providing friends and family with education is no longer supported (the strength of support was weak before) |  |
|  | **Willingness to participate** / Demographics / age | 1 | 1 | 0 | 0 | **17** | Higher age associated with higher utilization of behavioral health services was a weak factor associated with willingness to participate perspective. | This factor contributed to ‘support delivery’ factors of the **Employee (in)ability to talk about mental health and ask for help’** theme and although these factors are supported elsewhere, the removal of low-quality studies weaken this aspect of the themes. |
|  | Job role | 1 | 2 | 0 | 0 | **17** | A weak factor in the higher-level node of  ‘*Willingness to participate’*, weakened further by sensitivity analysis left unsupported by sensitivity analysis. The data in this node related to job title being associated with likelihood of recommending support to others. | This factor is not a stand-alone factor in the themes, therefore its removal had negligible effect. |
|  | **Willingness to participate/** Length of service / Newly qualified staff / Orientation to include MH awareness and training | 2 | 4 | 0 | 0 | **16, 29** | This was a small factor, but provision of such awareness & training made sense. | This information is now unsupported; however, this information was not included as a separate factor and does not affect the theme construction. |
| **Enablers Culture** | **Culture/** Support offered is inclusive and representative of the workforce population | 2 | 3 | 0 | 0 | **16, 29** | A small but important factor. We know from the psychology and organizational support literature that ED&I factors are key. | This data is unsupported following sensitivity analysis. The following item in the organizational section of the support theme is compromised by sensitivity analysis:  **Person-centered** support that is **tailored to job role** risk and is **inclusive and representative** of the workforce population.  This highlights one of the limitations of this review in that the voices of a diverse population may not be reflected in the data. |
|  | Culture/ **Organisational endorsement of the support provided/** Stigma Reduction/ Stigma reduction is cost and outcome effective | 1 | 1 | 0 | 0 | **16** | A small factor included in the child node of ‘*Stigma Reduction’*, the removal of this information has little impact on the ‘higher level *Enablers* /‘*Culture’* node. | This information is now unsupported; however, this information was not included as a separate factor and does not affect the theme construction. |
|  | Culture/ **Organisational endorsement of the support provided/** The Organization encourages and prioritizes uptake of employee support/ Raise awareness through the voices of those with lived experience | 1 | 4 | 0 | 0 | **29** | A small factor included in the child node of ‘*The Organization encourages and prioritizes uptake of employee support’*, the removal of this information has little impact on the ‘higher level ‘*Enablers* / *Culture’* node. | This information is now unsupported; however, this information was not included as a separate factor and does not affect the theme construction. Although the voices of lived experience are known to encourage others and normalize help-seeking behavior. |
|  | Culture/ Organisational endorsement of the support provided / **The Organization encourages and prioritizes uptake of employee support**/ Recognition and reward at work / Working environment and behaviors/ Recognition and reward at work | 1 | 1 | 0 | 0 | **16** | A small factor included in the child node of ‘*The Organization encourages and prioritizes uptake of employee support’*, the removal of this information has little impact on the ‘higher level ‘*Enablers* / *Culture’* node. | This information is now unsupported; however, this information was not included as a separate factor and does not affect the theme construction as factors such as reward and recognition are reflected elsewhere in the nodes and themes. |
|  | Organisational sensitivity towards employee work-related stress/ Integrated systems for identifying at risk staff/Implementation governance responsibilities and accountabilities | 1 | 5 | 0 | 0 | **16** | A small factor included in the child node of ‘*Integrated systems for identifying at risk staff*, the removal of this information has little impact on the ‘higher level ‘*Enablers* / *Culture’* node. | This information is now unsupported; however, this information was not included as a separate factor and does not affect the theme construction as factors such as policy and organizational strategy are reflected elsewhere in the nodes and themes. |
|  | Wellness checks | 1 | 7 | 0 | 0 | **16** | A small factor included in the child node of ‘*Integrated systems for identifying at risk staff*, the removal of this information has little impact on the ‘higher level ‘*Enablers* / *Culture’* node. | Unsupported information, which may be useful for framing supportive sessions. Wellness checks are specifically included as a factor in the themes, but would feed into factors of time at work, organizational support and staff disclosure. However, the removal of these references has negligible impact on the constructed themes. |
|  | Organisational sensitivity towards employee work-related stress/ Integrated systems for identifying at risk staff / Stakeholder engagement | 2 | 11 | 0 | 0 | **12, 16** | A small but important factor. This factor is important for informing decision-making about strategy, services and education an organization should offer, but is a weak factor in this review. | The following item in the organizational section of the culture theme is unsupported following sensitivity analysis:  **Stakeholder engagement** to inform decision-making. |
|  | Taking mental health seriously | 1 | 1 | 0 | 0 | **29** | This factor is woven throughout the review and the loss of one reference "*My company takes mental health seriously…I am proud of*  *my agency.*" Has negligible impact on the themes. | This information is now unsupported; however, this information was not included as a separate factor and does not affect the theme construction |
|  | **Culture /** Support offered is inclusive and representative of the workforce population | 2 | 3 | 0 | 0 | **16, 29** | A small but important factor. We know from the psychology and organizational support literature that ED&I factors are key. | This data is unsupported following sensitivity analysis. The following item in the organizational section of the support theme is compromised by sensitivity analysis:  **Person-centered** support that is **tailored to job role** risk and is **inclusive and representative** of the workforce population.  This highlights one of the limitations of this review in that the voices of a diverse population may not be reflected in the data. |
| **Enablers Staff** | Staff enablers / Disclosure / Speaking with peers / Choosing appropriate persons to provide peer support is key | 1 | 2 | 0 | 0 | **12** | A small factor in whether staff will talk to their peers – this factor is included in data elsewhere in the context of education. Therefore e.g., *The importance of choosing the appropriate peer support volunteers cannot be overstated, as this has already derailed more than one peer support program* has negligible influence on theme construction. | This information is now unsupported; however, this information was not included as a separate factor and does not affect the theme construction. |
|  | Staff enablers / Disclosure / Speaking with peers / Peer to peer screening for stress | 2 | 3 | 0 | 0 | **13, 29** | A small factor included in the child node of ‘*Disclosure’* the removal of this information has little impact on the ‘higher level ‘*Enablers* / Staff*’* node. | This information is now unsupported; however, this information was not included as a separate factor and does not affect the theme construction. |
| **Enablers Support** | Staff enablers / Proactive Support/ Proactive follow-up/ Follow-up undertaken by individuals not responsible for line management | 1 | 1 | 0 | 0 | **13** | A small factor included in the child node of ‘*Proactive support’* the removal of this information has little impact on the ‘higher level ‘*Enablers* / Staff*’* node. | This information is now unsupported; however, this information was not included as a separate factor and does not affect the theme construction. |
|  | Staff enablers / Research / Evaluation Measuring effectiveness / Quality Assurance | 1 | 1 | 0 | 0 | **12** | A small but important factor. This factor is important for informing decision-making about strategy, services and education an organization should offer, but is a weak factor in this review:  *‘Sharing of anonymized or aggregate information* *between and across paramedic service organizations with comparable data points and standardized measures could improve service delivery and potentially assist in reducing operational stress injuries’* | This data is unsupported following sensitivity analysis. The following item in the organizational section of the Training theme is compromised by sensitivity analysis:  **Evaluate and measure** the effectiveness of support provided. |
|  | Staff enablers / Research / Evaluation Measuring effectiveness / Sharing of anonymized data across ambulance organization’s | 1 | 1 | 0 | 0 | **16** |  |  |
|  | Evidence-informed interventions and appropriate support | 2 | 2 | 0 | 0 | **16, 29** | A small factor that is represented in the barrier’s themes.  *Though no one suggests that mental health issues can be fully prevented, advice often given by medical professionals (and backed up by research) is that there are some things people can do to protect their mental health.* | This information is now unsupported; however, this information is inversely reflected in the barrier themes and although the loss of this data weakens the following organizational factor in the Support theme:   - **Robust evidence-base** fostered through high-quality, context specific research.   Therefore, the removal of this data has a negligible impact on theme construction overall. |
| **Enablers Training** | Enablers/ Training / Training Staff/ Prevention and recognition of mental ill health training / Substance misuse education | 2 | 2 | 0 | 0 | **12, 29** | A small but important factor. We know from the psychology and organizational support literature that substance misuse in an important influencing factor, however the included articles did not focus on this issue as a barrier of facilitator to employee support.  *"The link between PTSD and substance abuse has been disregarded for a long time."*  *Some prevention training discusses inappropriate use and abuse of substances to manage stress through pre incident and post-incident education.* | Substance abuse was not included as a separate factor within the themes and this node supported the development of more general factors in the Training Theme such as   - Education for staff to **recognize symptoms** of acute and cumulative stress and distress in themselves and their colleagues.   Therefore, the removal of this data has a negligible impact on theme construction overall. |

Factors left unsupported following sensitivity analysis are highlighted in yellow
