# Supplementary figures and images for: Why Do Emergency Medical Service Employees (Not) Seek Organizational Help for Mental Health Support?: A Systematic Review
Source: Int J Environ Res Public Health. 2025 Apr 17;22(4):629. doi: 10.3390/ijerph22040629 (PMC12027444; doi:10.3390/ijerph22040629)

Supplementary Materials S5, Figure S1: PRISMA flow diagram


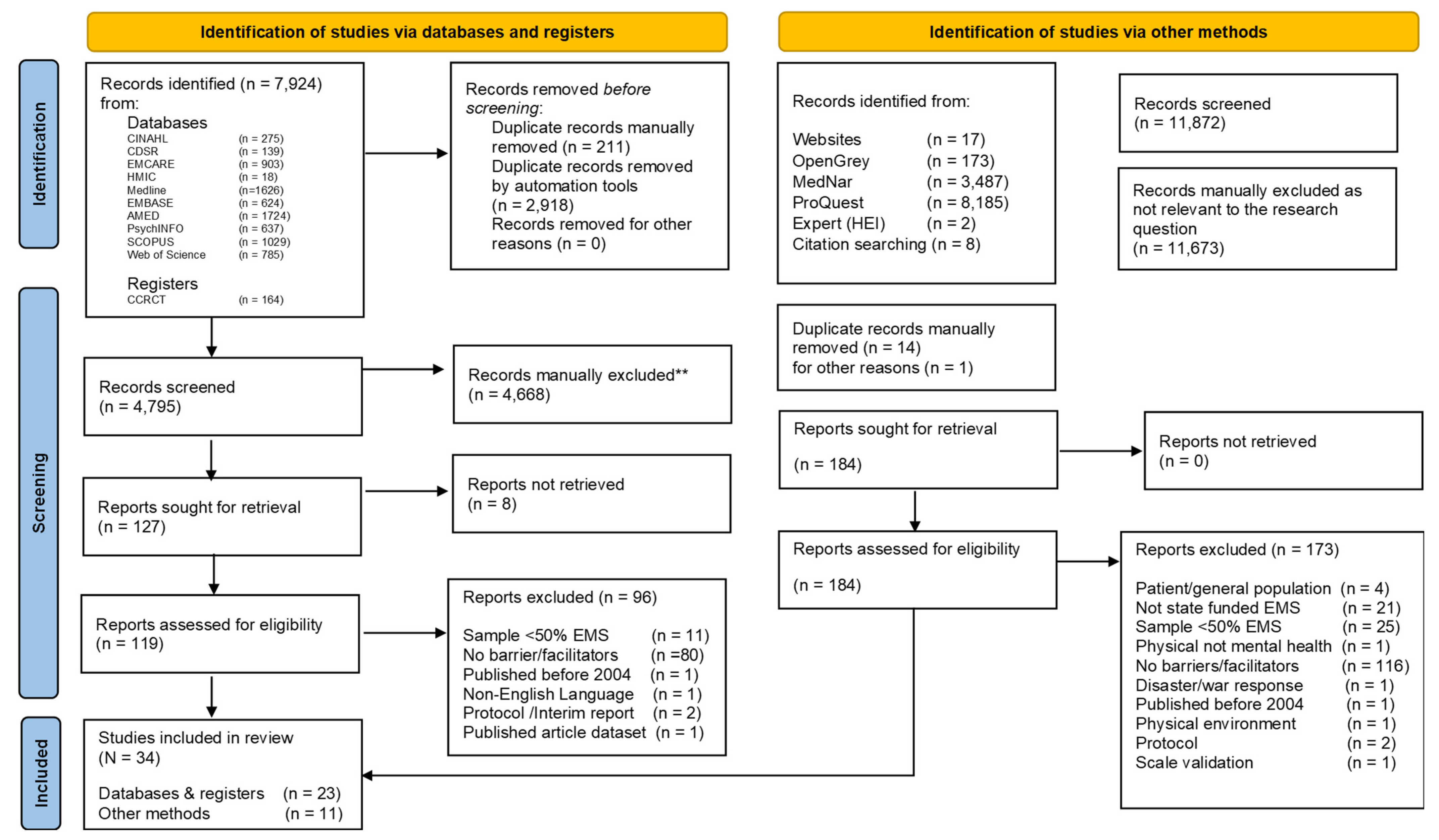

Supplement: Supplementary file 1 [file ijerph-22-00629-s001.zip › Supplementary Material S5—Figure S1 PRISMA flow diagram.docx]
